# Anaerobic cryoEM protocols for air-sensitive nitrogenase proteins

**DOI:** 10.1038/s41596-024-00973-5

**Published:** 2024-04-04

**Authors:** Rebeccah A. Warmack, Belinda B. Wenke, Thomas Spatzal, Douglas C. Rees

**Affiliations:** 1 Division of Chemistry and Chemical Engineering 147-75, California Institute of Technology, Pasadena, California 91125, United States; 2 Howard Hughes Medical Institute, California Institute of Technology, United States

## Abstract

Single particle cryo-electron microscopy (cryoEM) provides an attractive avenue for advancing our atomic resolution understanding of materials, molecules, and living systems. However, the vast majority of published cryoEM methodologies focus on the characterization of aerobically purified samples. Air-sensitive enzymes and microbes represent significant, yet understudied systems in structural biology. We have recently demonstrated the success of an anaerobic single particle cryoEM workflow applied to the air-sensitive nitrogenase enzymes. In this protocol, we employ a protein tag for monitoring sample exposure to oxygen in air, and detail the use of Schlenk lines and anaerobic chambers to prepare samples. We describe how to use a plunge freezing apparatus inside of a soft-sided vinyl chamber of the type we routinely use for anaerobic biochemistry and crystallography of oxygen-sensitive proteins. Manual control of the airlock allows for introduction of liquid cryogens into the tent. A custom vacuum port provides slow, continuous evacuation of the tent atmosphere to avoid accumulation of flammable vapors within the enclosed chamber. These methods allowed us to obtain high resolution structures of both nitrogenase proteins using single particle cryoEM. The procedures involved can be generally subdivided into a 4-day anaerobic sample generation procedure, and a 1-day anaerobic cryoEM sample preparation step, followed by conventional cryoEM imaging and processing steps. As nitrogen is a substrate for nitrogenase, the Schlenk lines and anaerobic chambers described in this procedure are operated under an argon atmosphere; however, the system and these procedures are compatible with other controlled inert gas environments.

## EDITORIAL SUMMARY

It can be challenging to obtain meaningful and accurate structural information for air-sensitive proteins. This protocol describes the application of customized vacuum-manifold and anaerobic chamber setups for the purification and cryoEM analysis of air-sensitive nitrogenase enzymes.

## Introduction:

Nearly half of the metabolically useful nitrogen is presently estimated to derive from biological nitrogen fixation via the enzyme nitrogenase^1^. Nitrogenase catalyzes the ATP-dependent reduction of atmospheric dinitrogen to ammonia through the reaction shown in Equation 1^2^.


(Equation 1) $$N_{2}+10H^{+}+8e^{-}+16ATP\to 2NH_{4}^{+}+H_{2}+16ADP+16P_{i}$$

This enzyme consists of two proteins, the obligate reductase Fe-protein and the catalytic MoFe-protein. Turnover is facilitated by electron transfer through the constituent metalloclusters of each protein, including the [4Fe:4S] cluster of the Fe-protein (FeP), the [8Fe:7S] P-cluster of the MoFe-protein (MoFeP), and the [7Fe:9S:1C:1Mo]-*R-*homocitrate FeMo-cofactor within the active site of the MoFe-protein (see collection of reviews edited by P. Holland^3^). The Fe- and MoFe-proteins are both inactivated upon exposure to oxygen, with the Fe-protein being the more sensitive of the two^4^. A number of intermediate states have been proposed since the first comprehensive kinetic investigation of the *Klebsiella pneumonia* nitrogenase proteins by Thorneley and Lowe^2,5^. While progress has been reported in the structural characterization of various liganded forms of the FeMo-cofactor^6–12^, on-path intermediates have proved difficult to visualize due to the high protein concentration and homogeneity required for canonical X-ray crystallographic techniques. The advent of single particle cryo-electron microscopy (cryoEM) and its increasing gains in resolution^13,14^ offer a potential avenue for the capture and characterization of these states^15–17^. However, samples for this method are typically prepared on commercially available benchtop plunge freezing robots. That methodology is incompatible with the oxygen-sensitive nitrogenase proteins.

In a recent study, we published the first anaerobically frozen single particle cryoEM structures of the nitrogenase MoFe-protein including structures of a turnover-induced inactivated state and in complex with a previously uncharacterized endogenous binding partner^15^. Here, we describe the application of an oxygen-sensitive fluorescent protein tag and the anaerobic methodology used for the purification and cryoEM analysis of the nitrogenase proteins.

### Development of the protocol

For decades, defined atmosphere experiments have been integral to the fields of anaerobic chemistry and biology. They have been enabled by air-free methodologies including vacuum-gas manifolds and controlled chambers to maintain anoxic environments for the study of air-sensitive samples^18^. The ability to work anaerobically opens research possibilities that are currently only performed by a small number of groups. With practice and experience, any manipulation that can be done aerobically can be done anaerobically.

A number of labs have developed specific methods for the anaerobic purification of the nitrogenase Fe- and MoFe-proteins^19–21^. These proteins have been isolated from a variety of organisms including both strict aerobes and anaerobes^22–24^, although the nitrogenase proteins from *A. vinelandii* remain the most well-characterized due to the genetic tractability of this organism and the ease of culturing this obligate aerobe^25,26^. Following lysis of these microbes, reported purification protocols typically involve the combined use of Schlenk lines and anaerobic chambers. The detailed methods may differ significantly between laboratories, including the use of affinity tags, choices of chromatography resins, and purification steps performed within or outside the anaerobic chamber^27,28^.

#### Internal oxygen-probe

**Fig. 1.**
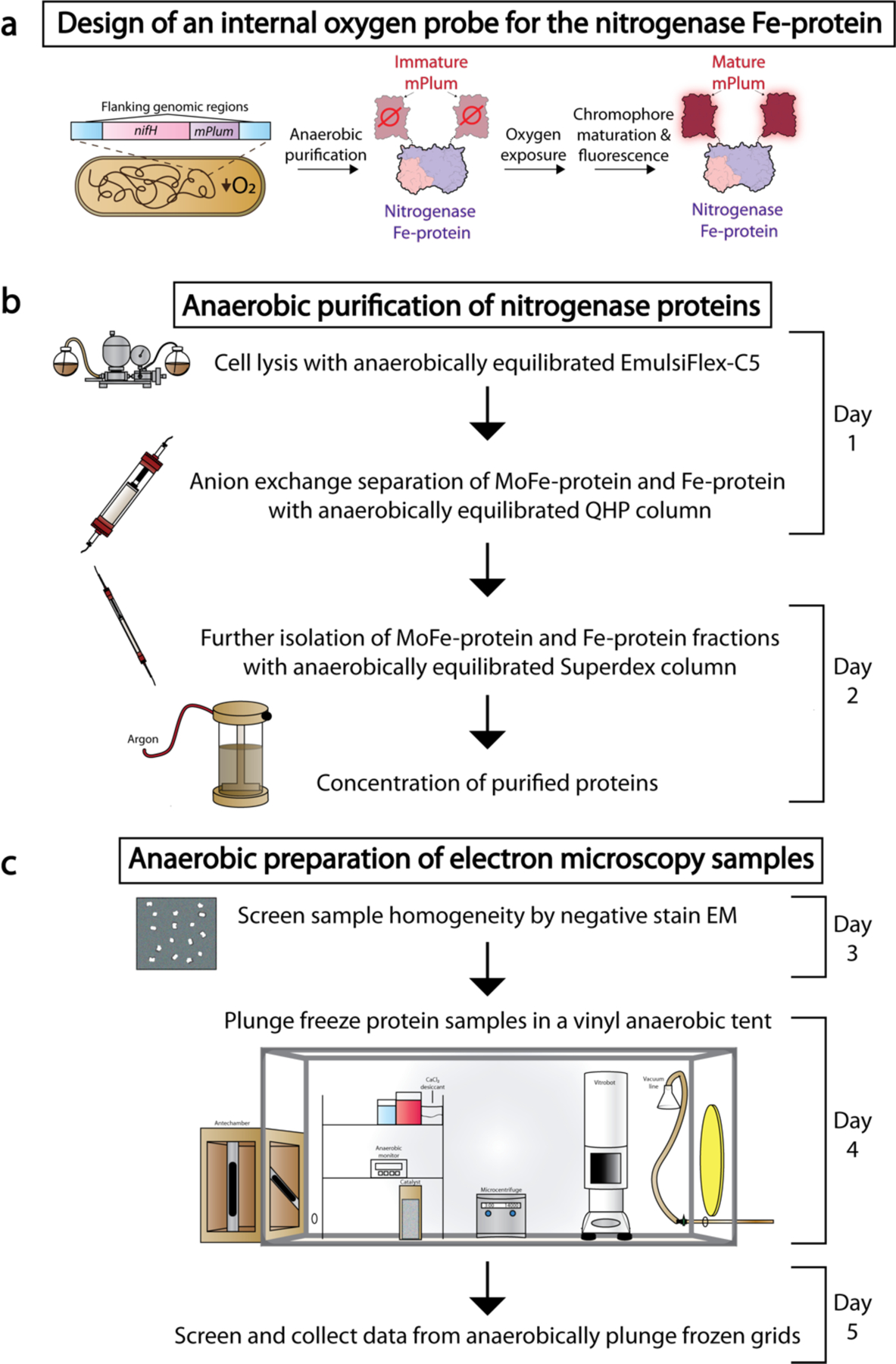
Overview of the procedure. (**a**) The diazotroph *Azotobacter vinelandii* was genomically edited to contain Fe-protein C-terminally tagged with mPlum. Within the reducing environment of the *A. vinelandii* cytoplasm, mPlum chromophore does not mature (immature mPlum). Once the protein is purified, if it is exposed to oxygen the mPlum chromophore will mature (mature mPlum) and can be visualized by eye if the aliquot turns purple, or through fluorescence at 649 nm. (**b**) Procedure part 1, schematic workflow of anaerobic purification of the nitrogenase proteins following a two-step procedure of anion exchange followed by size exclusion chromatography. **(c)** Procedure part 2, anaerobic preparation of samples for electron microscopy entirely within a vinyl anaerobic chamber or ‘tent’.

In all anaerobic methods, it is important to monitor any accidental exposure to oxygen that may compromise the chemical state of a sample. To this end, redox-active dyes such as resazurin, methylene-blue, and methylene viologen have been used as indicators when added to buffers and media for the detection of oxygen in cultures and purifications^29^. However, the use of these dyes may be suboptimal due to toxicity for users and may interfere with downstream applications. We have therefore engineered an internal oxygen-probe by genetically tagging the nitrogenase Fe-protein with the far-red fluorescent protein mPlum within *Azotobacter vinelandii*^30^ (Supp. Fig. 1). The tag is placed at the C-terminus of the Fe-protein, which is often disordered in crystal structures and is positioned remotely from the [4Fe:4S] cluster and the site of interaction with the MoFe-protein (Fig. 1a). This strain is able to grow robustly under nitrogen-limiting conditions, demonstrating that the construct is active and able to reduce atmospheric dinitrogen.

The chromophore of this protein tag requires molecular oxygen to mature, and can do so through one of two pathways, resulting in either a red or green chromophore^31^. During nitrogenase expression in nitrogen-limited cultures of *A. vinelandii*, the cytoplasm is maintained under reducing conditions through high rates of cellular respiration^32^. Thus, within these cells and upon successful anaerobic purification, the mPlum chromophore remains immature due to a low cytoplasmic concentration of molecular oxygen (Fig. 1). However, upon exposure of the purified protein to air, chromophore maturation can proceed and be monitored by fluorescence (excitation 590 nm, emission 649 nm). In consequence, the protein sample will visibly turn purple. This construct allows for visualization and quantification of oxygen exposure throughout the purification and all subsequent manipulations of the protein.

### Anaerobic protein preparation for cryoEM

The history of anaerobic techniques applied to the structural study of nitrogenase provides the foundation for the anaerobic single particle cryoEM methodology detailed in this paper. The sensitivity of the metalloclusters to oxidation and the consequent generation of heterogeneous degradation products necessitate a robust anaerobic protocol. Crystallization of the nitrogenase proteins has similarly been described within the glove box using either microcapillary or vapor diffusion methods^33^. However, given the need for specialized equipment associated with cryoEM procedures, including anaerobic chambers and plunge freezing robots, we first sought to test whether freezing anaerobic samples on a benchtop Vitrobot would be sufficient for maintaining reduced protein. Unfortunately, it became clear that grids frozen under even brief exposure to air (<~5 sec) showed partial oxidation of the P-cluster within the nitrogenase MoFe-protein^15^. Due to the high surface area of exposed grids, humidification of the blotting chamber and small volume handling, the use of chemical reductants was insufficient to protect the sample from oxidation. To control the atmosphere of the blotting process, an air-tight environment was required.

Vinyl Coy chambers have been used successfully for crystallization experiments, and a similar strategy was designed herein for grid preparation via the introduction of a ThermoFisher Vitrobot into a Coy vinyl tent. As liquid flammable cryogens are used for vitrification of the cryoEM grids, the Coy chamber was modified to include several ports fitted with copper vacuum lines to allow the evacuation of the tent atmosphere while the cryogen is inside the chamber. The explosion proof vacuum pump fitted to these lines has an outlet hose that vents into a fume hood for the safe exhaustion of any vaporized ethane/propane.

We developed protocols for use of this setup in the reproducible determination of high-resolution structures of both the nitrogenase Fe-protein and MoFe-protein and of the MoFe-protein:Fe-protein complex by single particle cryoEM. During the validation of this approach we discovered a strong propensity for MoFe-protein particles to adhere to the air water interface on grids, causing preferred orientation and denaturation of the protein^15,34^. We used previously published approaches^35–37^ including the application of surfactants and carbon-layered grids to successfully address this issue.

### Overview of the procedure

This protocol describes techniques and processes similar to those previously established for single particle cryoEM^38^, and overlaps with purification methods detailed for the tagged nitrogenase and other oxygen sensitive proteins^19,20,27,28^. However, the protocol also describes specific procedures involving the use of specialized anaerobic equipment tailored for cryoEM grid preparation, which require additional safety precautions. The Procedure is categorized into two sections taking place over five days: (1) the purification of the nitrogenase proteins, and (2) anaerobic preparation of single particle cryoEM grids (Fig. 1). Each step is described in further detail in the Procedures section.

#### The anaerobic laboratory

This procedure requires the physical space to house an anaerobic chamber, modifications to the anaerobic chamber, and requisite auxiliary equipment. The described laboratory consists of a single room with an area of less than 1,000 square feet. To avoid background reactions between nitrogen and nitrogenase in our experiments, the Schlenk lines and anaerobic chambers are maintained under 100% argon atmospheres and 95% argon:5% hydrogen atmospheres, respectively. Hydrogen is required for the scrubbing of oxygen from the tent atmosphere by the catalyst system in the anaerobic chamber. All gas tanks are chain-secured in metal storage racks, and all active tanks containing flammable gas are attached to regulators within a cabinet with a dedicated ventilation shaft. Copper tubing from the tanks is routed to the vacuum manifold or anaerobic chambers along grounded metal cable trays installed near the ceiling.

#### Custom vacuum-manifold set up

**Fig. 2.**
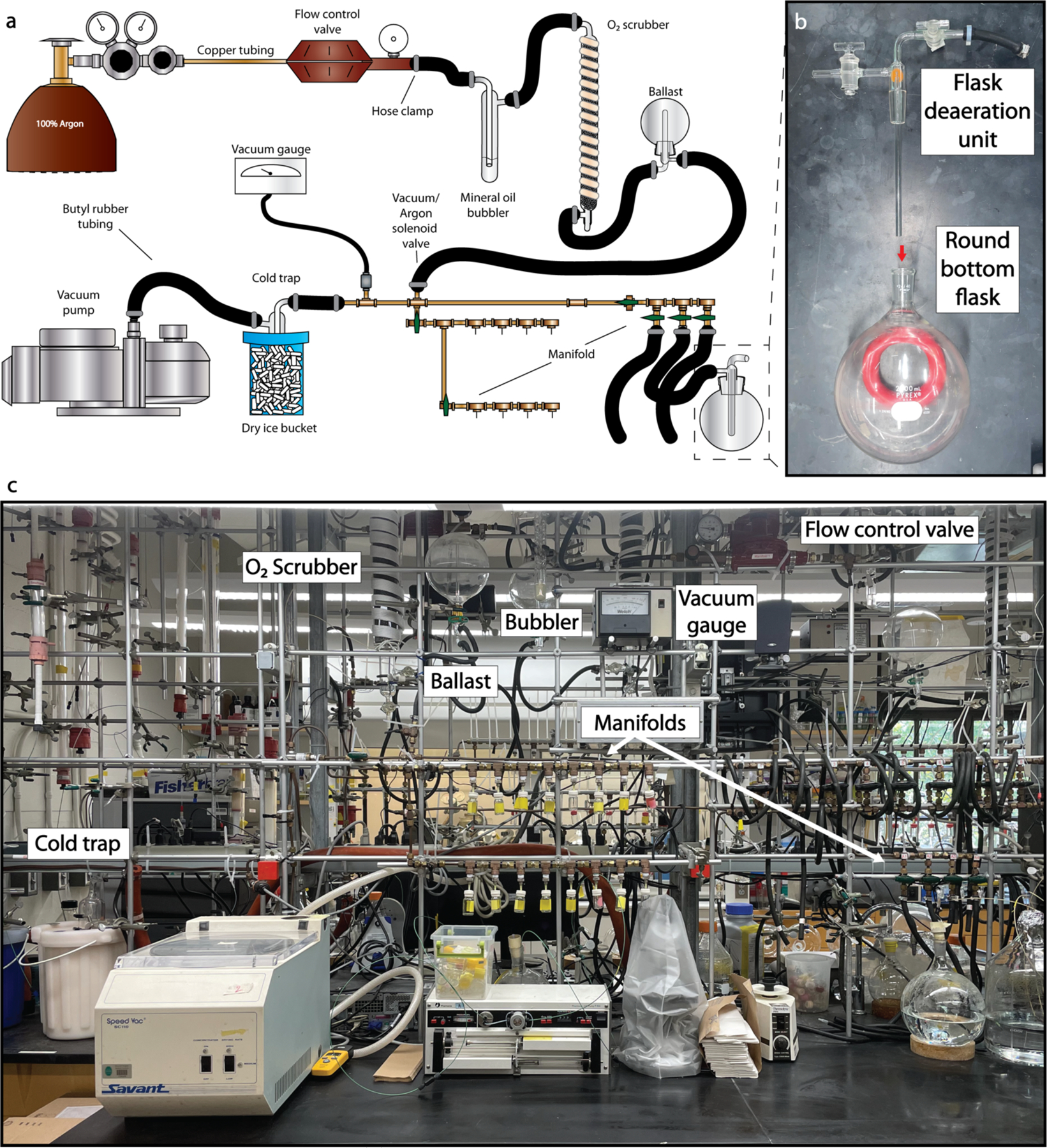
Custom vacuum-manifold set up. (**a**) Schematic diagram of custom built Schlenk line generally following flow of argon in a clockwise manner starting from the argon tank. (**b**) Assembly of the flask deaeration unit within the round bottom flask attached to the Schlenk line for degassing. (**c**) Image of the depicted custom built Schlenk line.

A vacuum-manifold is required for work involving anaerobic protein purification and anaerobic manipulation of solutions. Tubing to the manifold is routed to a low pressure line regulator, and any pressure fluctuations are buffered by a downstream glass ballast (Fig. 2). Glass mineral oil bubblers are installed inline to visualize any leaks within the system. In addition, to remove oxygen introduced through the tank or the lines, a custom oxygen scrubbing tower is installed which contains a copper catalyst. Argon flows through these checkpoints into a network of copper tubing connected by Swagelok joints and T-valves. These lines are also connected to hosing that can be attached to a cold trap kept in a dry ice ethanol bath, which is further connected to a vacuum pump. Vacuum within the lines is measured by a Welch vacuum gauge. This custom Schlenk line has outlet ports fitted with Luer-lock needles which can be pierced through rubber septa on smaller anaerobic flasks for direct deaeration. Needles are capped and these lines are closed when not in use. For larger volumes, separate outlet ports are attached to butyl rubber tubing that can be joined to a custom glass stem, termed a flask deaeration unit, fitted into the neck of a round bottom flask (Fig. 2b).

#### Customized vinyl anaerobic chamber

The anaerobic chamber – or tent – is required for all free-form benchtop manipulation of room temperature samples including the anaerobic preparation of cryoEM grids. In our configuration, a separate set of lines stemming from a 100% argon tank and a 95% argon:5% hydrogen tank lead to Coy vinyl anaerobic chambers, and these lines are threaded to the back of the airlock. The procedures we describe are feasibly accomplished within one anaerobic chamber and are described as such.

**Fig. 3.**
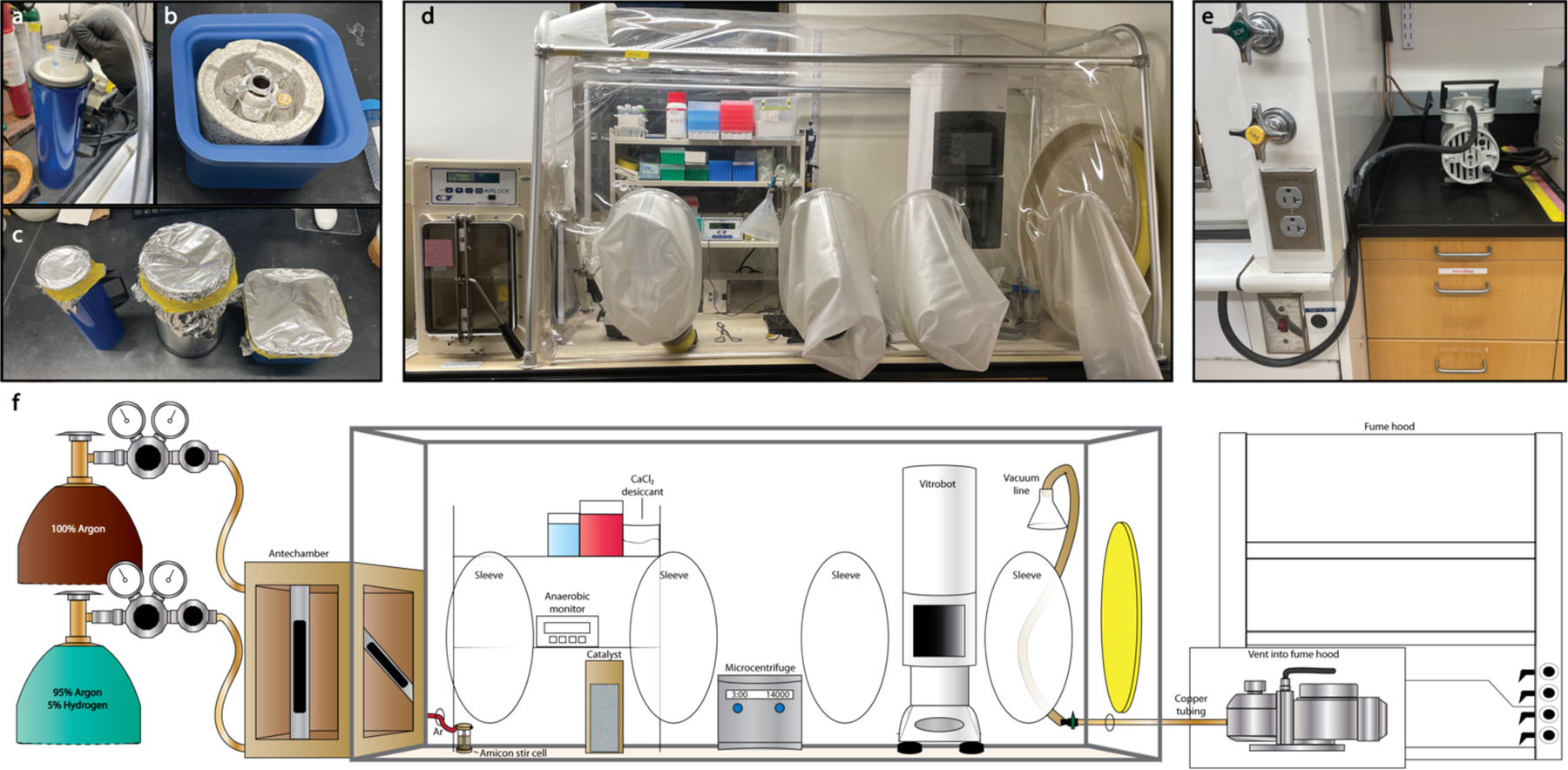
Infrastructure for anaerobic single particle cryoEM grid preparation. (**a**) Ethane/propane is dispensed within a 50 mL Falcon tube on liquid nitrogen. (**b**) The Vitrobot Dewar is assembled and placed within a secondary Dewar to capture any spilled cryogen. Before bringing into the tent the outer reservoir is filled with liquid nitrogen. (**c**) All Dewars to be brought into the tent containing cryogen are covered with aluminum foil secured with tape. (**d**) Customized vinyl anaerobic tent containing a ThermoFisher Vitrobot Mark 4. (**e**) Copper tubing is threaded out of the anaerobic tent to a vacuum pump which vents to the fume hood during grid preparation. (**f**) Schematic overview of setup for anaerobic single particle cryoEM grid preparation including illustration of customized ports for an Amicon stir cell for protein concentration and the port for atmospheric exhaust during grid preparation.

The chamber contains conventional accessories for single particle cryoEM experiments including pipets, pipet tips, a microcentrifuge, EM grids, filter paper, tweezers, forceps, and a plunge freezing apparatus. In addition, the chamber houses necessary elements for maintaining and monitoring the anaerobic atmosphere including catalysts, an anaerobic monitor, a resazurin solution^29,39^, and calcium chloride desiccant (Fig. 3).

To prevent the accumulation of ethane/propane vapors within the tent atmosphere during grid preparation, the chamber has a port containing a rubber stopper through which copper tubing has been threaded. This copper tubing is fitted with a T-valve within the tent to control access to the atmosphere. The other end of the tubing is connected to a vacuum pump that vents into a fume hood (Fig. 3e). The T-valve is partially opened and this vacuum pump remains in operation throughout the process of grid preparation. A second port can be threaded with a line that can be used to pressurize an Amicon stirred cell under argon for the anaerobic concentration of the nitrogenase proteins (Fig. 3f).

#### Protein Purification

The first section of this procedure details the application of the described equipment to the purification of the nitrogenase proteins from the obligate aerobe *A. vinelandii*. The procedure is applicable to both the endogenous proteins as well as those purified from a strain containing FeP-mPlum. The first two days of the experiment are spent preparing the equipment and reagents. As shown in Figure 1, protein purification typically takes place over two days; the process includes flash freezing of the samples and their storage in liquid nitrogen between steps. Purification can be accomplished within a single day if the protein samples are unstable upon isolation, as may occur with some mutant proteins.

Both Schlenk lines and anaerobic chambers are required for various steps to maintain an anaerobic state for the proteins during and after lysis. Specifically, lysis and purification steps are performed with equipment outside of the anaerobic chamber that has been equilibrated in de-aerated buffers; these remain connected to a flow of argon from the Schlenk line (Fig. 2). Lysis of cells, transfers of liquids, and protein concentration take place linked to a Schlenk line or within the anaerobic chamber (Fig.3). Small aliquots of the purified proteins are flash frozen and stored in liquid nitrogen.

#### Sample preparation for single particle cryoEM

The second section of the protocol describes the anaerobic preparation of single particle cryoEM samples from these purified proteins within the scope of 1 day. With the exception of cryogen preparation and glow discharging of the grids, this procedure takes place almost entirely within the anaerobic chamber (tent). All necessary stocks and materials are stored within the tent. Several steps are taken to safely transfer cryogens into the tent, as follows:

1. An ethane/propane mix is dispensed into a 50 mL Falcon tube partially submerged in liquid nitrogen. The cap is placed back on top of the tube, but the tube is not sealed to prevent trapping condensed oxygen (Fig. 3a).
2. To equilibrate the Vitrobot Dewar, the outer reservoir is filled with liquid nitrogen outside of the tent. To avoid spillover this Dewar is kept inside a secondary container (Fig. 3b).
3. All Dewars containing cryogens are covered with aluminum foil secured by tape (Fig. 3c).
4. Cryogens are cycled in through the airlock using a specific step-wise protocol.

### Application of the method

This Procedure is broadly applicable to the purification and structural cryoEM analysis of chemical or biological systems that require a controlled atmosphere. We have specifically applied the method to the oxygen-sensitive nitrogenase proteins, but these techniques can be directly translated to the study of other air-sensitive enzymes. These include metalloenzymes such as ribonucleotide reductases, hydrogenases, radical SAM enzymes, CO dehydrogenases, or for proteins with reduced cysteines where the thiol state is preferred over disulfide bridges^40^.

It should be emphasized that applications of this approach are not limited to single particle cryoEM. Once a plunge-freezing apparatus has been placed inside the anaerobic chamber, samples may be prepared for all other cryoEM modalities including electron diffraction (MicroED/3DED) and electron cryo tomography (cryoET). Microcrystals of air sensitive proteins, peptides, or small molecules may be grown and prepared within the anaerobic chamber, or even on grids^41^, and plunge frozen within the box without exposure to oxygen. Similarly, strict or facultative anaerobes may be cultured within the anaerobic chamber and cells applied to EM grids under controlled atmospheric conditions.

The techniques described within this article may be further adapted for sample preparation in a glove box for samples that may require more strict atmospheric control. CryoEM sample preparation within an anaerobic chamber also allows for exquisite control of atmospheric compositions allowing for the study of reactions or organisms under gas mixtures of varying concentrations or under partial vacuum.

### Comparison with other methods

The techniques described here derive from previously established single particle cryoEM protocols^38^ as well as purification methods detailed for the oxygen-sensitive proteins. However, there are significant differences with existing protocols in the use of specialized anaerobic equipment for the cryoEM grid preparation, which requires additional safety precautions, and the use of a novel nitrogenase construct using mPlum as an internal oxygen probe (Fig. 1). The vast majority of published single particle cryoEM structures have been frozen aerobically. The first description of the anaerobic single particle cryoEM method was provided in 2019^34^, based on methods established for anaerobic crystallography^33^. Recently, the Nicolet group detailed the application of anaerobic single particle cryoEM to air-sensitive protein samples^42^. In this study, procedures were outlined for bringing liquid propane and liquid nitrogen in the Vitrobot Dewar into a rigid anaerobic chamber. This was accomplished through airlocks connected to a gas-purification system and pressure regulator. While rigid chambers offer advantages, including lower gas permeability and higher resistance to organic solvents, many laboratories practicing air-free microbiology and biochemistry employ less expensive vinyl anaerobic chambers which expand and contract to maintain a more constant internal pressure than rigid chambers. They may also be manufactured on larger scales than rigid units, allowing for the internalization of larger pieces of equipment.

The partial solidification of liquid nitrogen described by Cherrier *et al*., is avoided in our procedure through serial cycling: a series of step-wise manual purging and evacuation within the tent’s antechamber. In addition, Cherrier *et al*. utilize 100% propane during vitrification, which also solidifies within the airlock during transfer. In the place of pure ethane or propane, we employ a 37% ethane/63% propane mixture as first described by the Jensen lab^43^. This mixture not only remains liquid during antechamber cycling, reducing wait times for liquification once the cryogen is cycled into the tent, but it was also found to reduce damage to carbon-layered grids. Accumulation of vapors from these cryogens within the tent atmosphere is prevented by slow, continuous evacuation of the chamber through a vacuum line during sample preparation.

Vitrification of samples within the anaerobic chamber follows a similar workflow to published aerobic methodologies^38^. A prerequisite to these experiments is the successful anaerobic purification of active nitrogenase proteins. Many recent publications that describe methods for the purification of nitrogenase utilize affinity tags on the MoFe-protein including Strep and His tags which provide robust preparations of active nitrogenase^19,20^. We have built upon this literature and produced an mPlum-tagged Fe-protein, wherein the maturation of the mPlum chromophore can be used as a visual marker of oxygen exposure. To eliminate artefactual biochemical and structural effects from exogenous tags, proteins purified from this strain were compared to purification of untagged, endogenous nitrogenase from the diazotrophic soil bacterium *Azotobacter vinelandii*.

While this manuscript was in preparation, a report appeared from Schmidt *et al*. describing cryoEM structures of the Fe-only nitrogenase prepared anaerobically within a Coy chamber^44^.

### Experimental design

Concentrations of 5 – 10 mM Na_2_S_2_O_4_ are used at several points throughout the protocol as a method for ensuring a highly reducing environment, particularly during points of the procedure at higher risk of oxygen contamination, for example in connections when hooking up the AKTA systems and the EmulsiFlex-C5.

#### Choice of Equipment Setup

Many laboratories working with oxygen sensitive samples utilize anaerobic chambers, and we anticipate that modifications to these existing systems can be devised to accommodate the described procedure through the introduction of a vitrification robot or manual plunge freezer, as well as the modification of a port within the anaerobic tent for atmospheric evacuation. We specifically describe the preparation of single particle cryoEM grids within a vinyl anaerobic chamber. Appropriate safety considerations must be taken into account when working with different glove box setups with distinct airlocks, such as a rigid glove box.

The main feature of our anaerobic cryoEM procedure is the housing of a plunge freezing robot, the Thermo-Fisher Vitrobot Mark IV, within a vinyl anaerobic chamber that has an additional port through which tubing attached to a vacuum pump can be threaded to vent tent atmosphere into a fume hood throughout sample preparation. Other commercial or custom-built plunge freezing apparatus may be used in place of the Vitrobot. Deoxygenated water and buffer stocks can be stored in the tent for Vitrobot equilibration and sample dilutions, respectively. To verify low oxygen levels within the atmosphere, an anaerobic monitor reporting oxygen levels should be housed within the chamber.

#### Considering the protein target

The Procedure focuses on purified protein samples from a non-pathogenic, BSL 1 soil bacterium. Researchers working with hazardous samples or pathogenic organisms must take additional safety precautions when designing their experimental set up.

The purification procedure would need to be modified depending on the organism and the protein or complex of interest. For example, the use of affinity tags could allow for more selective purification procedures, requiring specialized purification columns. Alternatively, with abundant proteins purified without a tag from their native source, cryoEM experiments could be conducted on a smaller scale and with less pure fractions. While the scale of bacterial cell growth and protein purification outlined here is sufficient to yield hundreds of milligrams of MoFe protein, as few as 100 μg of material are sufficient for cryoEM. In addition, certain air-sensitive metalloproteins may be purified aerobically before being subsequently reconstituted with the metal cofactors; these include certain nitrogenase associated proteins such as the radical SAM enzyme NifB^45,46^. Therefore in certain cases purification and grid preparation conditions may be optimized aerobically with the apo protein prior to attempting air-free procedures.

A number of proteins containing metal cofactors are oxygen sensitive, therefore it is additionally important to consider buffer components that may interact with metal ions or clusters during the experimental design stage^47^. Examples of these include Bis-Tris^48^ and ACES^49^ which interact with Cu(II). Alongside the buffering agent, the identity and concentration of salts present in the solution should be carefully considered. This is especially important in the case of nitrogenase, which exhibits decreased activity in solutions of increasing ionic strength^50^.

#### Checking for unintended oxidation

For proteins exhibiting redox dependent conformational changes, structural or spectroscopic analyses can help establish whether rigorously anaerobic conditions have been maintained. As an example, the nitrogenase P-cluster has a characteristic conformational change upon oxidation that can be observed at resolutions better than 2.5 Å. Thus, initial data collection and analysis of the resting state MoFe-protein can also be used to confirm that the anaerobic set up is maintaining the reduced form of the protein. This protocol involves the use of several instruments, and reagents to avoid oxidations. Sodium dithionite (Na_2_S_2_O_4_) is essential as a reducing agent in various buffers at several points throughout the protocol, since it maintains a highly reducing environment for proteins in solution when argon-purging is not possible. This is critical, for example, during use of protein purification instruments (AKTA systems) and cell lysis equipment (EmulsiFlex-C5).

Since neither the nitrogenase FeMo-cofactor nor the Fe-protein exhibit significant structural changes upon oxidation, an mPlum-tagged Fe-protein construct allows for monitoring of oxygen exposure throughout purification and grid preparation. The use of mPlum as an indicator may also be useful for other oxygen-sensitive enzyme systems that do not have a pronounced conformational change upon oxidation. While the mPlum-tag approach as outlined here is compatible with the nitrogenase system, it is important to consider the placement of the tag on the intended target and its potential interference with target activity when adapting the mPlum-tag approach for other proteins. For example, mPlum did not significantly alter the activity of nitrogenase as verified by our assays, and did not obstruct the overall distribution or orientation of Fe-protein targets on grids when freezing for cryoEM.

#### Choice of inert gas

While the nitrogenase system benefits from isolation and characterization in argon, to avoid the possibility of nitrogen exposure prior to biochemical or structural characterization, other protein systems may simply require a defined atmosphere, or specifically, oxygen avoidance. In such cases, an oxygen depleted atmosphere consisting primarily or entirely of nitrogen may be desirable to more closely mimic atmospheric compositions. This environment would be compatible with all equipment listed in this protocol and would facilitate oxygen-free work. It should be noted that the Coy anaerobic monitor that detects oxygen and hydrogen levels utilized within this protocol must be calibrated in the background of the desired inert gas. If this is not done, the newly introduced gas may be detected and read as hydrogen by the monitor. Other defined gases can also be introduced into the anaerobic tents, including controlled amounts of flammable gases, given the precautions outlined in this protocol.

It is worth commenting at this stage on the use of liquid nitrogen as a cryogen throughout the preparation of single particle cryoEM grids of nitrogenase and how this may affect interpretation of these structures. The equipment setup and grid preparation protocol described here are specifically designed to avoid accumulation of significant levels of evaporated cryogen through the use of continuous evacuation of the atmosphere and limiting the total time in which cryogens are kept in the tent, thus the levels of gaseous nitrogen found within the tent are anticipated to be low. In addition, protein samples are introduced to the grid within the Vitrobot and plunge frozen rapidly within liquid ethane/propane. Thus, when the sample is introduced to liquid nitrogen for storage the proteins are already frozen within a vitreous ice layer and are not capable of turning over substrate.

#### Oxygen is not the only source of structural artefacts

It should be emphasized that despite manipulation of samples within an inert atmosphere, the nitrogenase MoFe-protein is still subject to damage or partial denaturation at the “air”-water interface (AWI^15^). For any new protein sample, we strongly encourage screening of particle distribution within the ice using cryoET to eliminate the possible influence of the AWI on structural features^36,37^. The localization of nitrogenase to the AWI has dictated our grid types and conditions for cryoEM, including the use of carbon-layered grids or detergent, which may not be necessary for all air-sensitive protein samples.

#### Expertise required

In addition to general training in molecular biology and protein purification, this Procedure requires expertise in anaerobic biochemistry including manipulation of materials on Schlenk lines and working within anaerobic chambers. Methods involving changes in atmospheric pressure and closed systems, such as flasks, as well as using flammable cryogens within the anaerobic chamber should be approached with extreme caution as described throughout this protocol. Importantly, while the procedures described are designed to be carried out by a single person, some procedures, such as the growth and harvesting of cells could benefit from assistance with the handling of large culture volumes. Additionally, for steps where time is critical, such as the freezing of grids, certain steps such as grid glow discharging and work in the anaerobic tent can occur simultaneously if carried out by multiple people.

### Limitations of the method

The primary limitation of this procedure are the capital expenditures of specialized equipment that may not already be widely available in many laboratories starting work on air-sensitive samples. In addition, the precise manipulation of delicate samples in an anaerobic chamber can be cumbersome. This limited mobility can also pose safety risks when working with cryogens in the enclosed environment. This is particularly concerning with regards to spills, as liquid nitrogen has an expansion ratio of 1:694 (the ratio of the volume of the gas at room temperature to the corresponding volume of the liquified gas), and the flammable cryogens ethane and propane, have expansion ratios of 1:424 and 1:270, respectively. Due to the evaporation of the cryogens and concomitant dilution of hydrogen within the tent atmosphere, we suggest the total time course over which grids should be made within the box be limited to approximately 30 minutes in order to maintain efficient oxygen scrubbing by the catalysts. This time span can be extended by manually exchanging or ‘cycling’ the atmosphere in the tent to replenish hydrogen levels, however, this can be inconvenient in the middle of sample preparation. These factors impact the number of grids that can be made in a single session. Lastly, despite improved atmospheric control within the anaerobic tent over traditional grid preparation stations, typical problems associated with cryoEM sample preparation can still persist including protein denaturation at the air water interface, ice and ethane contamination, and uneven particle distribution.

## Materials:

### Biological materials

**! CAUTION** Users need to comply with their institutional biosafety policies.

*Azotobacter vinelandii* Lipman, strain designation OP (ATCC strain 13705). This wild-type strain was directly purchased from ATCC.

*Azotobacter vinelandii* FeP-mPlum, parent strain designation DJ54^51^. This strain was generated from the parent strain DJ54 (a kind gift from Dennis Dean) by introduction of the FeP-mPlum construct through homologous recombination. More information on the generation of the FeP-mPlum strain is available in Supplementary Figure 1.

### Reagents

- Ultrapure water, Milli-Q Advantage A10 Water Purification System (Millipore Sigma, cat. no. Z00Q0V0WW)
- Sodium hydrosulfite (Na_2_S_2_O_4_; dithionite; Sigma-Aldrich, cat. no. 71699–250G)
- Tris, ultrapure (MP Biochemicals, cat. no. 819620)
- Sodium chloride (Fisher Scientific, cat. no. S271-10)
- Hydrochloric acid (Sigma-Aldrich, cat. no. 320331-2.5L)
- 3-([3-Cholamidopropyl]dimethylammonio)-2-hydroxy-1-propanesulfonate (CHAPSO), anagrade (Anatrace, cat. no. C317 5 GM)

### Equipment

#### Inside anaerobic chamber:

- Customized type A Coy anaerobic chamber or ‘tent’ (Coy, cat. no. Type A)
- Anaerobic monitor (Coy Lab Products, CAM-12)
- Vitrobot Mark 4 (ThermoFisher Scientific, cat. no. VITROBOT)
- Standard Vitrobot Filter Paper, Ø55/20mm, Grade 595 (Ted Pella, cat. no. 47000–100)
- Cryo-Tweezers (NanoSoft, cat. no. 17021002)
- DeNovix DS-11 FX spectrophotometer/Fluorometer
- Pelco reverse fine tweezers (Ted Pella, cat. no. 5375NM)
- Formvar/Carbon 200 mesh, Copper (Ted Pella, cat. no. 01801)
- Quantifoil R1.2/1.3 300 Mesh EM grids, Copper (100/pk; Electron Microscopy Sciences, cat. no. Q3100CR1.3)
- Ultrathin Carbon on Quantifoil R1.2/1.3 300 Mesh EM grids, Copper (10/pk; Ted Pella, cat. no. 668–300-CU)
- Stak-Pak, catalyst (Coy Lab Products, cat. no. 6501000)
- Microcentrifuge (Eppendorf Centrifuge 5418, now 5418R, cat. no. 5401000137)
- Centrifuge bottles (Beckman Coulter, cat. no. 356011)
- Amicon concentrating filters (Millipore Sigma, cat. no. UFSC05001 (50 mL); cat. no. 5121 (10 mL))
- Ultrafiltration Discs (Fisher Scientific, cat. no. PLHK04310)
- Stir plate
- WHEATON^®^ Potter-Elvehjem Tissue Grinder, serrated pestle, 55 mL (DWK Life Sciences, cat. no. 357994)
- Digital scale (American Weigh Scales, cat. no. SM5DR)

#### Outside anaerobic chamber:

- Custom-built Schlenk line^18,52^
- Vacuum pump for Coy tent (Gast, cat. no. 1023-101Q-G608NGX)
- Pelco reverse fine tweezers (Ted Pella, cat. no. 5375NM)
- Cryo Grid boxes (Electron Microscopy Sciences, cat. no. 711166-110-W)
- PELCO easiGlow^™^ Glow Discharge Unit (Ted Pella, cat. no. 91000)
- ÄKTA Explorer FPLC pumps (Cytiva, now ÄKTA avant, cat. no. 28976337)
- Q Sepharose High Performance resin (Cytiva, cat. no. 17101401)
- XK 26/40 Column (Cytiva, cat. no. 28-9889-49)
- XK26 column adaptors (Cytiva, cat. no. 28-9898-77)
- HiLoad 26/600 Superdex 200 pg column (Cytiva, cat. no. 28989336)
- Centrifuge (Beckman Coulter Avanti J-26 XP, cat. no. B22987)
- JA-14 fixed angle rotor (Beckman Coulter, cat. no. 339247)
- EmulsiFlex-C5 (Avestin)
- Nitrogen aluminum freezer (Airgas, cat. no. MVB10719924)
- Assorted sizes of round bottom flasks (RBFs)
- Keck joint clamps (VWR, cat. no. 80061)
- Custom made flask deaeration units
- Custom made glass mineral oil bubbler
- Custom made glass oxygen scrubbing tower
- Custom made glass ballast
- Custom made glass solvent trap
- Vacuum pump (Edwards, cat. no. A65401906)
- Vacuum gauge (Welch, cat. no. 1515)
- Large rubber septa, 24/40 joints (Sigma-Aldrich, cat. no. Z553980)
- Small rubber septa, 7.9/14 joints (Sigma-Aldrich, cat. no. Z564702)
- 100% argon tank (Airgas, cat. no. UN1006)
- 95% argon 5% Hydrogen tank (Airgas, cat. no. UN1954)
- 37% Ethane/Propane tank (Airgas, custom mix)
- Welch vacuum pump model 2019B-01 (Fisher Scientific, cat. no. 12-584-506)
- Low pressure line regulator (Matheson, cat. no. 3702)
- Assorted Swagelock copper/bronze fittings
- ¼” copper capillary tubing (Grainger, cat. no. 66DC65)
- Butyl rubber tubing (Fisher Scientific, cat. no. 12–168B)
- Dow Corning high vacuum grease (Fisher Scientific, cat. no. 50-955-8411)
- Wheaton glass serum bottle, volume 50 mL (Sigma-Aldrich, cat. no. Z113999)
- Wheaton glass serum bottle, volume 100 mL (Sigma-Aldrich, cat. no. Z113999)

### Reagent setup

**! CAUTION** Handle glass flasks under vacuum with extreme care, blast shields and secondary containers may be used to mitigate risk.

#### Anaerobic ultrapure water stock

Degas 1 L ultrapure water, 18.2 MΩ-cm from an ultrapure water purification system in a 2 L round bottom flask (RBF) using 12 cycles of 7 min vacuum followed by 2 min argon purge. After degassing, pull vacuum on the flask for 7 min. Close valves and cycle this solution into the tent through the antechamber of the anaerobic tent. Stored at room temperature, within the tent, this stock has indefinite shelf life, but should be evaluated periodically for contamination.

#### Anion exchange (AEC) wash buffer

Prepare a solution with the following composition:

50 mM Tris-HCl, pH 7.8
150 mM NaCl

After degassing as described below, just before use, add solid Na_2_S_2_O_4_ to a final concentration of:

5 mM Na_2_S_2_O_4_

Separately degas 1 L each in two 2 L RBFs with 12 cycles of 7 min vacuum followed by 2 min argon purge. After degassing, pull vacuum on one of the round bottom flasks (RBFs) for 7 min. Close stopcocks on the flask deaeration unit and cycle this RBF into the tent through the antechamber of the anaerobic tent. Leave the remaining flask under argon on the Schlenk line. Stored at room temperature while connected to the Schlenk line, this buffer should be degassed again if not used within two days to ensure anaerobicity.

#### AEC elution buffer

Prepare a solution with the following composition:

50 mM Tris-HCl, pH 7.8
1 M NaCl

After degassing as described below, just before use, add solid Na_2_S_2_O_4_ to a final concentration of:

5 mM Na_2_S_2_O_4_

Degas 1 L in a 2 L RBF with 12 cycles of 7 min vacuum followed by 2 min argon purge. After degassing, leave the flask under argon on the Schlenk line. Stored at room temperature while connected to the Schlenk line, this buffer should be degassed again if not used within two days to ensure anaerobicity.

### Size exclusion chromatography (SEC) buffer

Prepare a solution with the following composition:

50 mM Tris-HCl, pH 7.8
200 mM NaCl

After degassing as described below, just before use, add solid Na_2_S_2_O_4_ to a final concentration of:

5 mM Na_2_S_2_O_4_

Separately degas 2 L each in two 4 L RBFs, as well as an additional 1 L in a 2L RBF, using 12 cycles of 7 min vacuum followed by 2 min argon purge. After degassing, pull vacuum on the 2L RBF for 7 min. Close valves and cycle this solution into the tent through the antechamber of the anerobic tent. Leave the remaining flasks under argon on the Schlenk line. Stored at room temperature while connected to the Schlenk line, this buffer should be degassed again if not used within two days to ensure anaerobicity.

### CHAPSO stock

Prepare a solution with the following composition:

1% *w/v* CHAPSO
50 mM Tris-HCl, pH 7.8
200 mM NaCl

Weigh out the necessary amount of CHAPSO on the benchtop within a 10 mL Wheaton vial. Sparge the vial with argon from the Schlenk line. Cycle this Wheaton vial into the tent through the antechamber of the anaerobic tent. In the tent, use the degassed SEC buffer to prepare the 1% CHAPSO stock. Make a fresh CHAPSO stock for each experiment.

#### Equipment setup

##### Custom-built Schlenk line

Run ¼” copper tubing from the gas tanks to laboratory bench area where the Schlenk line is mounted on a vertical scaffold. First install an inline pressure reducer, followed by a mineral oil bubbler to detect leaks, an oxygen scrubbing catalyst, and ballast in order (Fig. 2). Downstream of these elements use a three way connection to join the copper tubing with tubing stemming from a vacuum pump, with a cold trap, and vacuum gauge. Store argon tanks chained in metal racks with regulator.

##### Customized vinyl anaerobic tent (Coy Vinyl Anaerobic Chamber, Type A)

The vinyl tent should be fitted with two additional ports (Fig. 3). One port is threaded with copper tubing to connect to the vacuum pump. The second port is threaded with tubing to the Amicon stirred ultrafiltration cell which is connected via a three way valve to the copper tubing extending from the argon tank. During installation of the tent, the Thermo-Fisher Vitrobot, or an equivalent plunge freezing apparatus, and any other large equipment should be carefully placed inside the tent through the side port prior to sealing and degassing. The Vitrobot foot pedal is placed inside the anaerobic tent with the rest of the machine and is operated by hand during sample preparation.

##### EmulsiFlex-C5 for anaerobic cell lysis

**Fig. 4.**
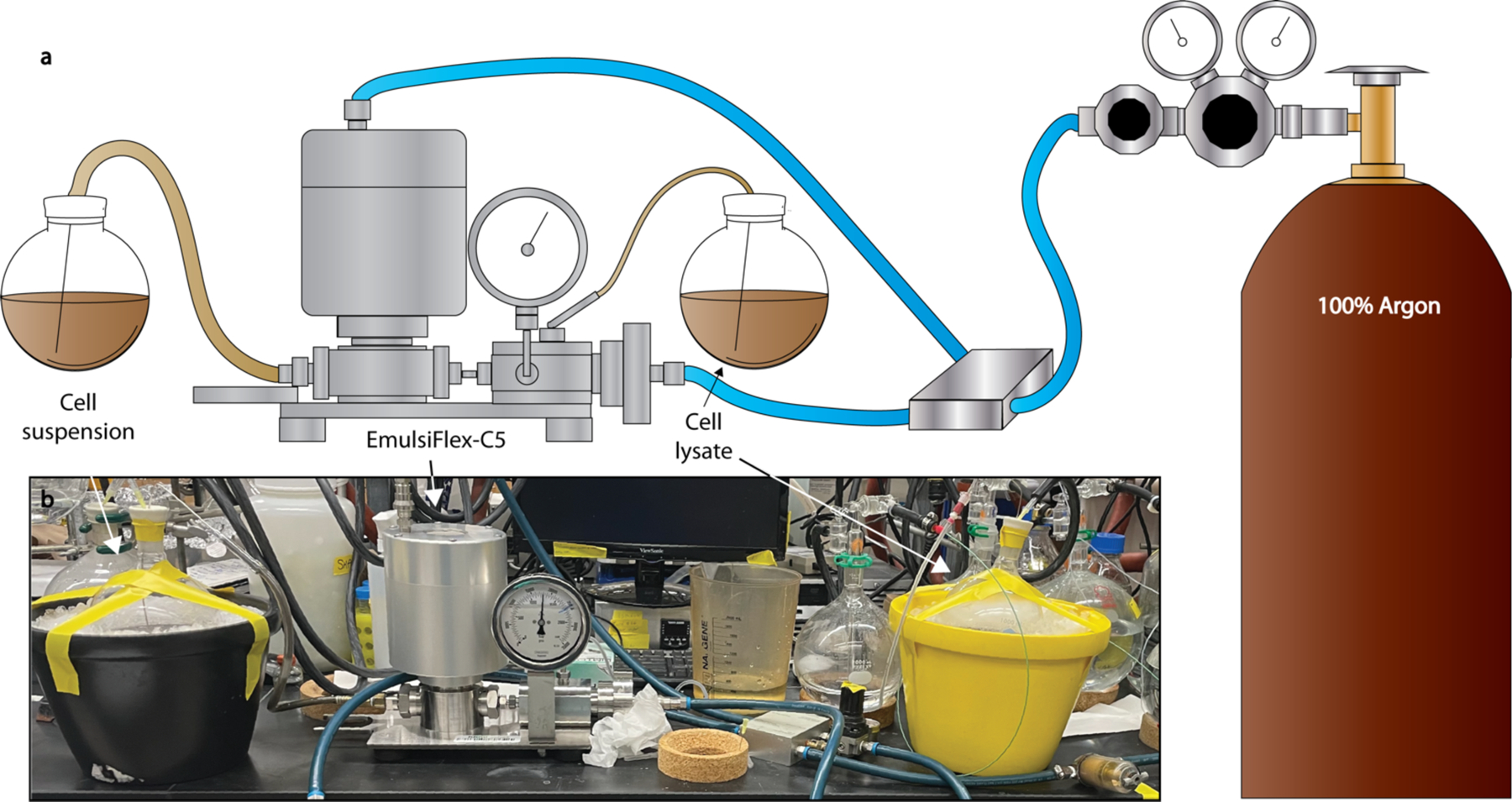
Overview of anaerobic cell lysis. (**a**) Schematic of EmulsiFlex-C5 connected to argon and anaerobic round bottom flasks. (**b**) Image of equivalent set up in lab. RBFs are connected to argon via the Schlenk line.

The EmulsiFlex-C5 air supply hose should be connected to an argon gas tank, and the remaining lines assembled following the manufacturer’s instructions. The sample cylinder can be removed and replaced with an equivalent fitting connected to Tygon tubing with a Luer-lock syringe and bent needle. Outlet tubing should also be fitted with a needle (Fig. 4). During lysis, anaerobic solutions in RBFs, including the AEC wash buffer, resuspended cells, and resulting lysate, should be sealed with rubber septa, kept on ice, and kept under argon from the Schlenk manifold. Inlet and outlet tubing can be connected to these solutions by piercing the rubber septa with the needles extending into the solutions. Lysis can then proceed as described by the manufacturer’s instructions. See Procedure for more information about subsequent steps.

##### ÄKTA Explorer FPLC pumps for anaerobic protein purification

FPLC pumps should be installed following the manufacturer’s instructions. The ÄKTA tubing should be replaced with PEEK tubing due to the low oxygen permeability. Outlet PEEK tubing from the FPLC can be fitted with a needle via a piece of Tygon tubing with an inner diameter slightly larger than the outer diameters of the PEEK tubing and needle. During anaerobic equilibration of pumps and columns, the inlet PEEK tubing is attached to de-aerated buffer solutions by screwing the fitting to the custom flask deaeration units (Fig. 2). During sample loading from anaerobic flasks sealed with rubber septa, the fitting from the inlet PEEK tubing can be removed and replaced with a needle as described for the outlet tubing. These inlet needle can then be inserted into the anaerobic sample flask.

##### Q Sepharose High Performance anion exchange column for nitrogenase protein purification

For a typical purification of nitrogenase proteins from approximately 50 g *A. vinelandii* cells, our lab employs 70 mL Q HP columns. This volume of resin is chosen so that it will be in excess of all potentially bound protein in a 50 g pellet of cells at pH 7.8, however, other column volumes may be used if found to be optimal. Assemble column and column adaptors, and pack column following the resin manufacturer’s instructions taking care not to exceed the maximum pressure limit of the resin. We suggest packing the column in the AEC wash buffer.

## Procedure:

### Anaerobic purification of Mo-nitrogenase MoFe- and Fe-proteins • TIMING 4 days (including preparation)

#### Day 1 (Setup and column equilibration):

1. Move all consumables necessary for the protein purification into the anaerobic tent.
2. Attach a SEC column to the FPLC, which is outside of the anaerobic tent. Begin washing the SEC column with 1 L H_2_O (aerobic) to remove the ethanol storage solution. Set UV monitors to 280 nm and 410 nm. Run overnight.
3. Attach an AEC column to a second FPLC, outside the anaerobic tent. Begin washing the AEC column with 500 mL ultrapure H_2_O (aerobic). Set UV monitors to 280 nm and 410 nm. Run overnight.

#### Day 2 (Buffer preparation and column equilibration):

- Attach a cold trap to a Schlenk line manifold; cool with a dry ice and ethanol bath. Turn on the vacuum pump attached to the Schlenk line.
- Prepare water, AEC wash buffer, AEC elution buffer, and SEC buffer as described in Reagent Setup and dispense into appropriate RBFs.
- Use vacuum grease to lubricate the flask deaeration units, then place these units into the necks of the RBFs containing buffer solutions.
- Secure each RBF neck with a clamp.
- Close the stopcock to the buffer line and attach butyl rubber hosing from the Schlenk line to the argon inlet stem of the flask deaeration unit (Fig. 2a–b).
  **! CAUTION**: To prevent liquid build up within the manifold, only degas a maximum of three large buffer solutions at one time.
- Degas buffer solutions with 12 cycles of alternating vacuum evacuation (7 min) and argon purge (2 min).
- Degassed solutions can be stored on the benchtop attached to an argon line until use.
- For buffer solutions that are to be brought into the anaerobic tent, after the final cycle, apply a vacuum for 7 min, close the argon inlet stem of the flask deaeration unit, place into the antechamber of the tent and cycle.
  **! CAUTION**: If the RBF is not placed under partial vacuum, during automated cycling within the antechamber when vacuum is pulled, positive pressure within the flask can cause the flask to explode.
- Attach degassed SEC and AEC buffers to respective FPLC buffer lines. To avoid trapping air within the lines follow these steps:
  - Connect the buffer solutions to the Schlenk line. Make sure the stopcock to the argon inlet is open. When opening, visually confirm argon is flowing, indicated by the bubbling of argon through the mineral oil bubbler.
  - Hold a thumb over the buffer outlet line of the flask deaeration unit and then open this stopcock.
  - Slowly release the thumb. Slight argon overpressure from the Schlenk line will push the buffer solution upwards through the primary stem.
  - When buffer solution has breached the buffer outlet line, attach the FPLC tubing.
- Begin equilibrating SEC and AEC columns in 1 L SEC buffer and 500 mL AEC wash buffer, respectively. Run overnight.
- After degassing buffer solutions, turn off vacuum pump attached to the Schlenk line, release residual vacuum from lines, and remove lines from the cold trap. Clean and dry the trap.

#### Day 3 (Cell lysis and AEC separation of nitrogenase proteins):

**Fig. 5.**
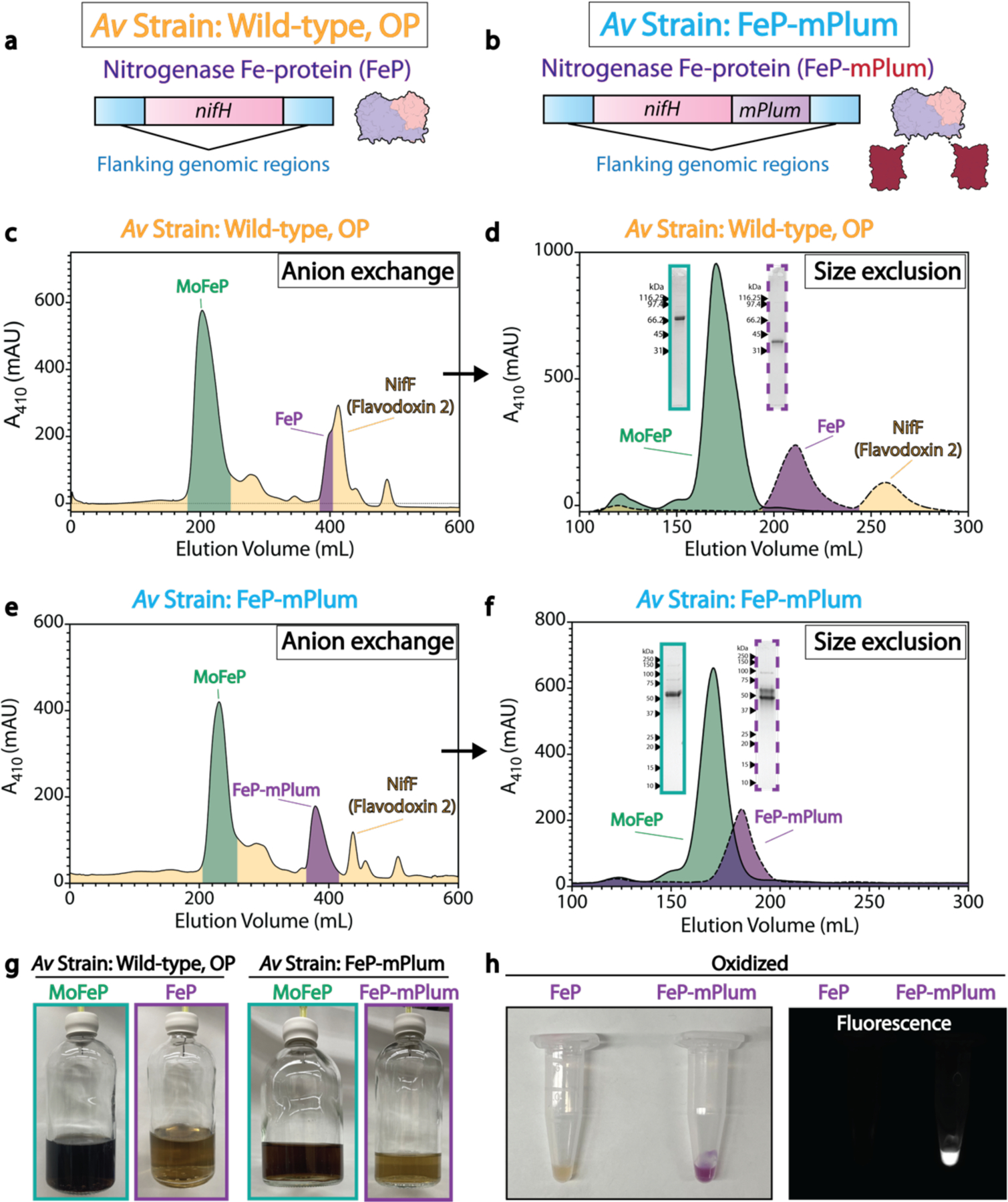
Purifications of nitrogenase proteins from the wild-type and mPlum-tagged strains. (**a-b**) Description of strains utilized for nitrogenase expression. (**c**) AEC chromatogram of the nitrogenase MoFe-protein (MoFeP) and Fe-protein (FeP) from the wild-type *A. vinelandii* strain. (**d**) SEC chromatograms of the nitrogenase proteins from the wild-type *A. vinelandii* strain. Insets display the gel of the purified proteins. (**e**) AEC chromatogram of the nitrogenase MoFeP and Fe-protein-mPlum (FeP-mPlum) from the mPlum-tagged Fe-protein *A. vinelandii* strain. (**f**) SEC chromatograms of the nitrogenase proteins from the FeP-mPlum *A. vinelandii* strain. Insets display the gel of the purified proteins. (**g**) Fractions of the MoFe- and Fe-proteins from size exclusion chromatography. (**h**) Oxidized aliquots of the Fe-protein from the wild-type and FeP-mPlum *A. vinelandii* strains. Right panel shows the fluorescence of the oxidized mPlum tag at 647 nm.

- Set up the EmulsiFlex-C5 as described in the Equipment Setup section (Fig. 4). Equilibrate with 200 mL ultrapure H_2_O (aerobic).
- Add 5 mM Na_2_S_2_O_4_ to AEC buffers, equilibrate column with 5 column volumes (CV) of buffer solution. Na_2_S_2_O_4_ absorbs strongly at 280 nm and therefore equilibration of the column can be monitored by elution of Na_2_S_2_O_4_ off the column at 280 nm.
- Place JA-14 rotor into Beckman Coulter Avanti J-26 XP centrifuge and let cool to 4 °C.
- Degas approximately 8 Wheaton bottles (4 each of the 50 and 100 mL bottles) for fraction collection on the Schlenk line using 12 cycles of vacuum (2 min) and argon (1 min). The number of Wheaton bottles used depends on the number of peaks to be sampled. Eight bottles allow for sampling of the principal peaks containing nitrogenase components and other potential peaks of interest.
- Introduce and gas exchange 50 g of *A. vinelandii* cell pellet into the antechamber of the EmulsiFlex-C5.
- Add Lysis Buffer (AEC wash buffer with 10 mM Na_2_S_2_O_4_ freshly added) at a ratio of 9.5:1 mL to g cells (475 mL for 50 g cell) in a glass beaker. Stir the solution on a plate for 20 min or until solution is homogeneous.
- Using a tissue grinder and serrated pestle, further homogenize the cell suspension in 50 mL aliquots. Dispense homogenized aliquots into a 1 L RBF. Once the cell suspension has been fully transferred, cap RBF with a large rubber septum.
- Prepare 300 mL of an anaerobic EmulsiFlex-C5 flush solution (AEC wash buffer with 50 mM Na_2_S_2_O_4_ freshly added). Place into a new 1 L RBF and cap with a large rubber septum.
  **▲ CRITICAL STEP** It is important to use a buffer that contains sodium dithionite (Na_2_S_2_O4) at this step as a reducing agent. As previously indicated, concentrations of Na_2_S_2_O_4_ are essential at several points throughout the protocol as a method for generating a sufficiently reducing environment when argon-purging is not possible, for example in connections when hooking up the AKTA systems and the EmulsiFlex-C5.
- Cap an empty 1 L RBF with a large rubber septum for the collection of lysate.
- Remove the cell suspension, the flush solution, and the empty RBFs from the tent and attach to argon lines on the manifold.
- Connect the flush solution to the EmulsiFlex-C5 as described in the Equipment Setup section and flush with 100 mL of de-oxygenated AEC wash buffer to make anaerobic and equilibrate into the buffer solution.
- Connect the *A. vinelandii* cell suspension to the EmulsiFlex-C5 and lyse at 25,000 psi by passing the solution once through the instrument, pressurizing with argon. Collect lysate into the empty RBF on ice.
- After lysis, cycle the RBF containing the lysate back into the anaerobic tent, dispense the contents into Beckman Coulter 250 mL centrifuge bottles, and balance these bottles using a scale. Cap bottles using lids with O-rings to create an air-tight seal. Remove the bottles from the tent.
  **! CAUTION** Be careful not to overfill round bottom flasks with cell lysate during lysis. Introduction of large volumes into the enclosed round bottom flask will create an overpressure. During cycling in the airlock, over-pressurized flasks can explode.
- Centrifuge to remove the cell debris at 30,000g for 45 min at 4 °C.
- After spin, cycle centrifuge bottles back into the anaerobic tent. Decant supernatant into a new 1 L RBF. Cap with a large rubber septum.
  **▲ CRITICAL STEP** Be careful not to disturb the pelleted debris. Cell debris in the solution can clog the AEC column in later steps.
- Separately prepare 50 mL fresh 5 mM Na_2_S_2_O_4_ solution in AEC wash buffer within a 100 mL Wheaton bottle. Cap with a small rubber septum.
- Remove the RBF containing the clarified lysate and the 5 mM Na_2_S_2_O_4_ buffer solution from the anaerobic tent and attach to argon lines to maintain anaerobicity. At this stage, the lysate is typically dark brown to black in color.
- If AEC column is finished equilibrating in Na_2_S_2_O_4_-containing buffer, pause the pump, close the buffer outlet stopcock of the AEC wash buffer RBF, and detach the wash buffer line.
- Insert a cannula needle with tubing slightly larger than the diameter of the FPLC PEEK tubing into the 5 mM Na_2_S_2_O_4_ buffer solution and allow argon overpressure to fill the cannula with anaerobic buffer.
- Transfer the anaerobically-purged cannula to the clarified lysate solution. Insert the end of the FPLC PEEK tubing into the rubber tubing of the cannula while avoiding air into the lines.
- Once attached, load lysate onto the AEC column at 5 mL/min, do not exceed 0.3 MPa pressure.
- Once the lysate is fully loaded, pause the FPLC pump, remove PEEK tubing from the cannula and reattach to the degassed AEC wash buffer flask as described in Day 2, Step 12.
- Wash column with AEC wash buffer until baseline is reached, continue to wash for an additional CV.
- Separate proteins using a gradient of 0% to 100% AEC elution buffer at a flow rate of 5 mL/min for 13 CVs. Figure 5 shows a characteristic chromatogram trace of the eluted fractions. Individual peaks are identified by A_410_ absorbance and corresponding fractions are manually collected in sealed anaerobic Wheaton bottles for subsequent activity assays (Fig. 5c,e). The MoFe- and Fe-proteins elute within the indicated fractions which are characteristically a dark and light brown, respectively. Their presence in these fractions can confirmed by SDS-PAGE, enzymatic activity and mass spectrometry.
- Collect MoFe- and Fe-protein fractions in degassed 100 mL Wheaton vials.

##### TROUBLESHOOTING

- Cycle protein fractions into anaerobic tent.
- Use the 50 mL Amicon filtration unit with a 100 kDa MWCO filter to concentrate the MoFe-protein fractions, and a 30 kDa MWCO filter to concentrate Fe-protein fractions to <10 mL volume.
  **▲ CRITICAL STEP:** Do not exceed 35 psi pressure within the Amicon filtration unit to avoid protein precipitation and leaking of the filtration unit.
- Transfer concentrated protein fractions to 15 mL Falcon tubes (do not exceed 5 mL volume in each tube). Remove from anaerobic tent and immediately flash freeze in liquid nitrogen.
  **! CAUTION** Liquid will expand during flash freezing within the falcon tubes, do not overfill falcon tubes to avoid cracking. Further, use polypropylene tubes to avoid shattering upon expansion during freezing.
- Exchange falcon tube caps with caps containing a drilled or punctured hole approximately 3–5mm in diameter (achieved using a power tool or other device), so that tubes will remain submerged and filled with LN2. The tubes can be stored within a liquid nitrogen Dewar.
  **■ PAUSE POINT** Aliquots frozen under these conditions can be safely stored at liquid nitrogen temperatures for an extended period of time (months).
- Add solid Na_2_S_2_O_4_ to SEC buffer to a final concentration of 5 mM and equilibrate SEC column with 1000 mL of the solution overnight.

#### Day 4: SEC purification of MoFe- and Fe-protein AEC fractions

- Degas approximately 8 Wheaton bottles for fraction collection on the Schlenk line using 12 cycles of vacuum (2 min) and argon (1 min).
- Transfer SEC FPLC buffer line to a fresh RBF flask of SEC buffer. Add 5 mM Na_2_S_2_O_4_ final concentration to fresh buffer solution.
- Cycle frozen MoFe-protein AEC fractions into anaerobic tent. Allow to thaw, filter, and transfer filtrate into Wheaton bottle. Cap with small rubber septum.
- Remove the MoFe-protein AEC fraction from anaerobic tent and attach to argon line.
- Use cannula as described in Day 3, steps 17–21 to load sample onto SEC column at 2 mL/min (do not exceed 0.3 MPa).
- Elute protein from column at 2 mL/min. Collect peaks in degassed Wheaton vials (Fig. 5d,f,g).
- Perform steps 47–50 for the Fe-protein AEC fractions.
- Concentrate fractions to <5 mL using the 10 mL Amicon filtration unit with a 100 kDa MWCO filter to concentrate the MoFe-protein fractions, and a 30 kDa MWCO filter to concentrate Fe-protein fractions. The MoFe-protein fractions should be a dark brown, while the Fe-protein fractions are typically lighter brown in color (Fig. 5g).
- Remove aliquots of final protein fractions for determination of protein concentration, analysis by SDS-PAGE, and specific activity in the acetylene reduction assay^53^.
- (Optional) If using the mPlum-tagged Fe-protein strain, aliquots may also be taken for fluorescence measurements to assess possible oxygen exposure using the DeNovix fluorometer. The DeNovix fluorometer should be kept inside the anaerobic tent for the analysis.
- Flash freeze remaining protein in liquid nitrogen and store in Dewar until use.
  **■ PAUSE POINT** Aliquots frozen under these conditions can be safely stored at liquid nitrogen temperatures for an extended period of time (months).

##### Negative stain screening of nitrogenase EM grids (OPTIONAL) • TIMING 1 – 4 h

**<CRITICAL>** While this step is optional, it can be useful to perform negative stain EM to confirm that protein complexes have assembled before proceeding to cryoEM analysis.

- Glow discharge 300 or 400 mesh copper grids covered with a carbon only or carbon-coated formvar film. Optionally, for improved contrast during imaging, use grids containing a lacey carbon film covered with 3 nm ultra-thin carbon.
- Within the tent apply 3 μL of a 0.01 mg/mL protein solution to grid. Wait 45 sec.
- Remove excess with blotting paper.
- Immediately apply 3 μL of a 1% uranyl acetate solution. Wait 45 sec.
- Remove excess with blotting paper. Air dry.
- Image sample on grids using a low energy 100 or 200 KeV (FEI T12) electron microscope. Imaging is typically performed at low magnifications (1,000x – 10,000x) to assess overall protein dispersity and concentration, and at higher magnifications (70,000x – 100,000x) to assess the overall quality of individual particles or protein complexes. Automated surveying tools such as SerialEM^54^ can be used to atlas entire grids, grid squares, or select areas within a grid. With a dataset containing a few thousand particles, one may achieve a recognizable reconstruction between 10–20 Å resolution.
- Obtain initial reconstructions of negative stain particles to assess purity and successful complex formation.

##### Anaerobic preparation of nitrogenase single particle cryoEM grids

###### • TIMING 1.5 h

- Cycle frozen nitrogenase protein aliquot into tent.
  **! CAUTION** If using cryogenic tubes, liquid nitrogen can enter and expand upon tube thawing causing the tube to explode. Make sure to vent tubes by untwisting cap immediately after transferring out of the airlock into the anaerobic tent.
- Set up Vitrobot as described by the manufacturer using degassed ultrapure H_2_O stock stored in anaerobic tent. Allow the Vitrobot enclosure to equilibrate to the desired humidity and temperature.
- Prepare a fresh solution of 5 mM Na_2_S_2_O_4_ in degassed SEC buffer solution stored in the anaerobic tent.
- If freezing MoFe-protein on grids, prepare a 1% CHAPSO stock using the 5 mM Na_2_S_2_O_4_ SEC buffer solution as described in the Reagent Setup section, in addition to a 4 mg/mL solution of MoFe-protein or nitrogenase complex. Alternatively, Quantifoil grids with ultrathin carbon can be used. If freezing grids of the Fe-protein, no CHAPSO solution is necessary.
- Spin down protein and CHAPSO solutions at 14,000 rpm for 3 min within a microcentrifuge inside the anaerobic tent. Transfer supernatants to fresh tubes.
- Using tweezers transfer four R1.2/1.3 grids from the manufacturer’s box to a clean microscope slide inside a glass Petri dish. Be careful not to damage the grids.
- Remove Petri dish with grids from the anaerobic tent and place microscope slide within the Pelco easiGlow glow discharge unit. Do not glow discharge yet.
- Fill a narrow 1 L Dewar, a 2 L Dewar, and the outer reservoir of the Vitrobot Dewar with liquid nitrogen. To prevent ice contamination when not in use, keep Dewars covered with aluminum foil.
- Within the narrow Dewar, equilibrate a 50 mL Falcon tube to temperature without allowing liquid nitrogen to enter the Falcon tube (Fig. 3a).
- Once the Falcon tube has reached the temperature of the liquid nitrogen, condense 10 mL of the 37% ethane/propane mixture into the Falcon tube. Lightly place cap back onto falcon tube, do not screw shut, keep tube partially submerged in the liquid nitrogen.
  **<CAUTION>** Do not completely close Falcon tube, as there is a risk of trapping highly reactive, condensed oxygen within the tube which cannot then be evacuated during manual cycling into the tent.
  **<CAUTION>** Consider the total volume of your anaerobic tent and the volume of flammable cryogen being brought inside. The lower explosion limit (LEL) of ethane is 3% and propane is 2.1%. The Coy Type A vinyl anaerobic tent has a volume of 1,200 L; thus, if 10 mL condensed ethane or propane were to completely vaporize and expand 200–400 fold it would still be well below the LEL of either cryogen. However, if smaller anaerobic tents or larger volumes of cryogen are to be used these calculations should be repeated to ensure safe ventilation of the flammable vapors.
- Cover the Dewars with a double layer of aluminum foil and tape around the perimeter (Fig. 3c).
- Enter manual cycling mode in the Coy antechamber interface.
- Place Dewars within the antechamber of the tent and manually cycle cryogens into the tent using a step-wise ramp-up regime with 0.5” Hg units until 10” Hg units is reached (i.e. 0.5” Hg - purge, 1” Hg - purge, 1.5” Hg - purge, etc.). After 10” Hg is reached, continue for 20 cycles of 10” Hg vacuum and argon purges, followed by 4 cycles of 10” Hg vacuum and argon/hydrogen purges. Cryogens may then be brought into the tent.
  **CRITICAL STEP** It is important to perform sufficient cycles to exclude oxygen from the airlock.
  **<CAUTION>** Pulling vacuum on liquid nitrogen lowers the boiling point. If the system is evacuated too rapidly, large volumes of liquid nitrogen can boil, evaporate, and expand rapidly within the small, enclosed space of the antechamber. The step-wise regime described here allows liquid nitrogen to slowly evaporate throughout the cycling without significant boiling off. If the aluminum foil covering the Dewars starts to dome and significant evaporation is observed, immediately open the outer door of the antechamber to allow release of the expanding vapors. Once the system has stabilized, manually cycling can be restarted.
- Once the cryogens have been placed inside the anaerobic tent, immediately turn on the vacuum pump attached to the outlet line from the tent as described in Equipment Setup. Open the stopcock on this line inside the anaerobic tent to allow evacuation of any vaporized cryogen during grid preparation.
  **<CAUTION>** Extreme caution must be exercised once cryogens are within the tent. A spill could result in rapid expansion of vaporized cryogen, damage to the tent material or other equipment within the tent. If a spill occurs, open the inner door of the antechamber and manually pull vacuum on the tent atmosphere to evacuate expanding vapors. If a very large spill occurs, the inner and outer doors can be opened to completely release expanding vapors.
- Outside of the tent, start glow discharging of the grids. Once glow discharging has finished, use the automated cycling interface of the Coy antechamber to cycle grids into the tent.
- Within the tent, remove tape securing the aluminum foil to the Dewars. Use liquid nitrogen from the 2 L Dewar to top off the outer reservoir of the Vitrobot Dewar.
- Using forceps, grab the 50 mL Falcon tube containing the ethane/propane mixture and dispense into the central reservoir of the Vitrobot Dewar.
- Carefully transfer the Vitrobot Dewar onto the ethane lift of the Vitrobot and use forceps to remove the Vitrobot Spider.
- For grid preparation of MoFe-protein, mix together 2 μL of 4 mg/mL protein stock and 2 μL of 1% CHAPSO solution and immediately apply to grid, blot, and plunge freeze.
- Grids may be frozen using desired blot settings which vary between Vitrobots. Liquid nitrogen may be topped off in the outer reservoir of the Vitrobot Dewar as needed.
  **CRITICAL STEP** The CAM-12 anaerobic sensor registers cryogen vapors released from liquid nitrogen as hydrogen. The vacuum pump allays, but does not completely prevent the accumulation of these vapors, which dilute hydrogen within the tent atmosphere, and diminish the efficiency of the catalysts in reducing oxygen levels in the tent. To avoid this buildup of both nitrogen and ethane/propane vapors in the tent and to avoid ice contamination, we suggest that the number of samples prepared at one time be limited between 4–8 grids. If more time is required for grid freezing, the tent atmosphere may be cycled during grid preparation. If necessary during this time period, the Vitrobot Spider may be replaced periodically to ensure that the ethane/propane mixture remains cold enough for vitrification.
  **<CAUTION>** Take care when manipulating the Vitrobot tweezers. Piercing of the fingers may result in injury and can create holes in the gloves of the anaerobic tent, compromising the anaerobicity of the atmosphere.
- After grids are frozen within the grid box, carefully place all Dewars back into the anaerobic antechamber. Close inner door and remove Dewars from the tent.
- Store grid box containing frozen grids within a liquid nitrogen Dewar.
- Cycle antechamber and then cycle the atmosphere of the tent in order to remove vaporized ethane/propane and nitrogen from the atmosphere. The external vacuum line used during grid preparation may be closed, and the vacuum pump turned off. A flammable gas detector may be placed inside the tent to indicate whether the tent has been sufficiently purged with 100% Ar.
  **■ PAUSE POINT** Frozen grids may be stored at liquid nitrogen temperatures for an extended period of time (months).

##### Cryo-screening of nitrogenase EM grids • TIMING 1 – 2 h

- Clip grids and transfer into the microscope^55^.
- Collect low magnification atlases of each grid.
- Based on low magnification atlases, identify a grid for further screening and load.
- Collect two dimensional images at high magnification within the grid holes to assess particle distribution and ice quality. Generally, particles will be evenly distributed throughout the grid hole, with ice thin enough to allow for sufficient contrast in visualizing particles. Ideally, particle orientations will be noticeably different at this stage, but can be more critically assessed after 2D class averaging. Conditions that lead to preferred particle orientations on the grid should be avoided.
  **▲ CRITICAL STEP** Collect a tilt series of the grid hole for cryoET to ensure that particles are not adhered to the air water interface.
- Collect images overnight for a preliminary reconstruction.
? TROUBLESHOOTING

## Troubleshooting

Troubleshooting advice can be found in Table 1.

## Anticipated results

**Fig. 6.**
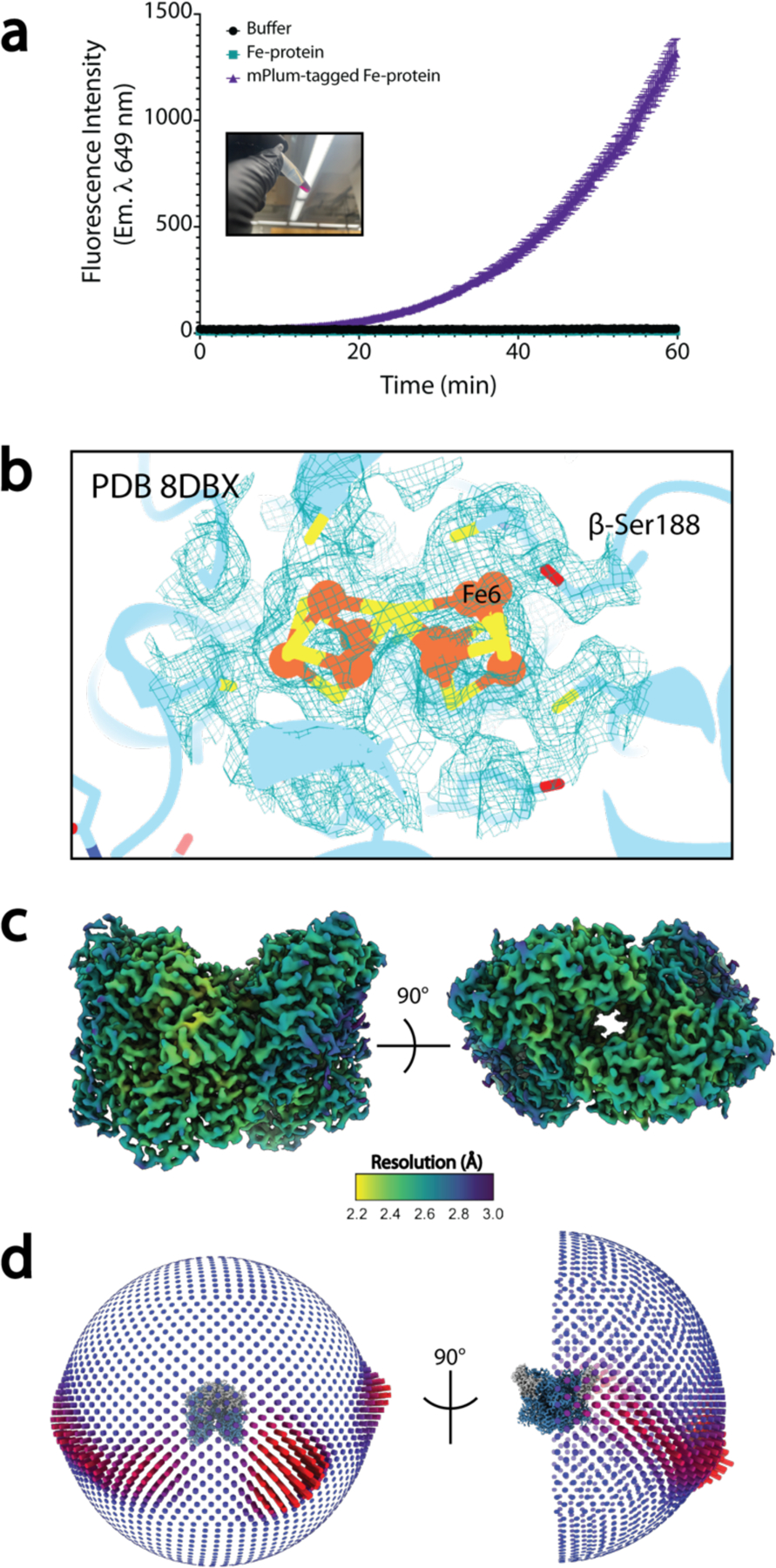
Potential pitfalls or causes for troubleshooting. **(a**) Observation of oxidized nitrogenase protein as indicated by fluorescent mPlum signal at 649 nm, or its colorimetric change due to oxygen exposure. Error bars shown are the standard deviation of measurements from three replicates. (**b**) Observation of P-cluster density in cryoEM maps of the nitrogenase protein, consistent with P-cluster oxidation. (**c**) CryoEM maps of nitrogenase indicating selective disorder in particular regions, indicative of possible air-water interface-induced damage (adaptation of Supp. Fig. 2a from ref. 14). (**d**) Bias in the orientation of identified nitrogenase particles as indicated in orientation distribution maps during 3D cryoEM reconstruction.

It is critical that throughout all of the Procedures described here, both the MoFe-protein and the Fe-protein of nitrogenase are kept anaerobic. Utilizing the mPlum-tagged Fe-protein construct, anaerobicity throughout this protocol can be assessed by removing aliquots of the purified protein and measuring fluorescence (Step 54; Fig. 6a). For the endogenous, untagged proteins the addition of oxygen-sensitive dyes to buffers and the use of anaerobic monitors within the tent may be employed instead. The conformation of the P-cluster of the MoFe-protein provides a sensitive indication of the oxidation state (Fig. 6b)^57^.Successful purification of active nitrogenase protein can be assessed by the specific activity as assayed by the reduction of acetylene substrate to ethylene^53^.

**Fig. 7.**
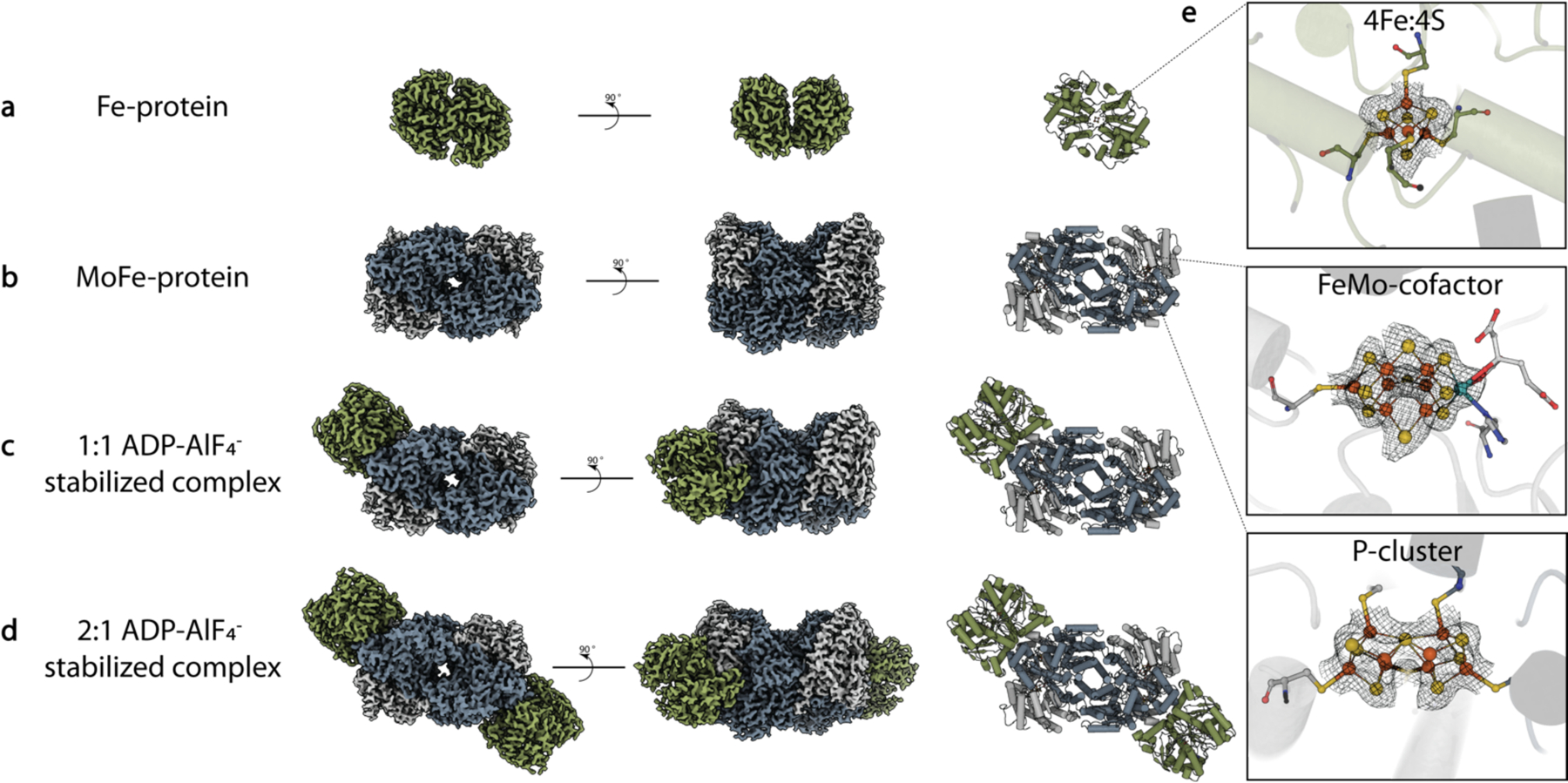
CryoEM structures of the nitrogenase Fe-protein, MoFe-protein, and ADP-AlF_4_^−^ stabilized complex states. (**a**) 2.6 Å resolution cryoEM map and model of the nitrogenase Fe-protein (mPlum-tagged; PDB code 8TC3). (**b**) 2.3 Å resolution cryoEM map and model of the nitrogenase MoFe-protein on ultrathin carbon (PDB code 8DBY). (**c**) 2.12 Å resolution structure of the 2:1 complex. (**d**) 2.48 Å resolution structure of the 1:1 complex (PDB code 8DFC). (**e**) Representative density for the metalloclusters (PDB code 8DFD).

To assess the success of the outlined anaerobic methodology for both the MoFe-protein and the Fe-protein, we prepared grids as described in Steps 63 through 85 and have collected single particle cryoEM data for the individual nitrogenase proteins as well as the ADP-AlF_4_^−^ stabilized complex of the two proteins (Fig. 7a–d; Supp. Fig. 2–4). These datasets have yielded a 2.6 Å resolution structure of the Fe-protein (mPlum-tagged), a 2.3 Å resolution MoFe-protein structure, and from samples of ADP-AlF_4_^−^ stabilized complex, a 2.12 Å resolution structure of the 2:1 Fe-protein:MoFe-protein complex, and a 1:1 complex at 2.48 Å resolution (PDB codes: 8TC3, 8DBY, 8DFD, and 8DFC, respectively). Due to the flexible nature of the linker between the Fe-protein and the mPlum tag, the mPlum tags were not resolved in the final structure, however, the core Fe-protein model correlates strongly with previously solved X-ray crystallography nucleotide-free structures, with an RMSD of 0.69 Å^2^, and clear density is observed for the 4Fe:4S cluster (Fig. 7e). Within the MoFe-protein and complex structures, the P-cluster of appears to be fully reduced supporting the robustness of the anaerobic freezing methodology presented here.

It should be noted that without the use of either detergent-containing conditions or carbon-layered grids as described in Step 66, substantial damage to the MoFe-protein was observed within the α-subunit during grid preparation (Fig. 6c–d). This is likely due to interactions with the air-water interface (AWI), as MoFe-protein particles were observed to localize to the AWI by cryoET. It is possible that, despite the use of carbon-layered grids, the observed 1:1 complex presented here is, in part, due to disruption of the interactions between the two proteins by residual competing interactions with the AWI, or from the shear forces of grid blotting within the Vitrobot or possibly the result of negative cooperativity between the two MoFe-protein dimers as proposed previously^16,58^. Further studies will help clarify the effects of these physical forces on protein complex integrity. Given the impact of additives on sensitive protein complexes, screening of other potential additives may be needed to ensure proper integrity of the determined structures.

## Supplementary Material

1

## Figures and Tables

**TABLE 1|:** Troubleshooting table

Step	Problem	Possible Reason	Solution
39, 51	A color change and fluorescence is observed in the Fe-protein-mPlum fractions during purification, requiring revisions to anaerobic technique starting at step 1 (Fig. 6a).	Exposure of the sample to oxygen due to: Insufficient deaeration of buffers. Gas permeable points in purification set up. Contaminating oxygen in anaerobic chambers or ‘tents’.	Add redox active dye, such as resazurin (1 mg/mL), to buffers to verify anaerobicity. Perform additional degas cycles as necessary. Running buffers through the columns without sample may be useful to ensure the setup is functioning as intended. Make sure all FPLC tubing is PEEK tubing. Fully equilibrate FPLC system with anaerobic buffer. Then run anaerobic buffer containing redox active dye through FPLC and collect fractions anaerobically (attached to the manifold). If a color change is observed, verify there are no leaks in the system by measuring flow rate. Check that the anaerobic monitors read below 10 PPM oxygen. In case of a faulty monitor, bring in a degassed aliquot of redox active dye and check for color change. If high oxygen levels are confirmed, then bring in fresh catalysts and cycle the entire antechamber to lower oxygen levels.
89	Too many, too few, or irregularly distributed particles on a grid, requiring re-optimization of grid conditions with the same protein preparation, returning to step 63 (Fig. 6d).	Suboptimal particle distribution due to: Too high or too low protein concentration. Protein aggregating in solution or on grid, leading to inhomogeneous spreading over grid. Protein preferring carbon over vitreous ice, or accumulating at the edges of holes.	Verify protein concentration using amino acid analysis, and prepare a set of grids with a range of different protein concentrations using the same blotting parameters. Adjust centrifugation settings of protein samples prior to grid preparation, including longer spin times and higher speeds. If aggregation is still observed on the grid, other surfactants besides CHAPSO, such as n-dodecyl-β-maltoside or fluorinated octyl-maltoside can be explored to minimize particle self-association^56^. Varying surfactants and blotting conditions, or using graphene-oxide, ultrathin-carbon layered, or UltrAUfoil grids may help establish an even distribution of particles across grid holes.
89	Contamination on surface of cryoEM grids, requiring revision to grid blotting, storage, or transfer setup, starting at step 63.	Crystalline ice may accumulate on grids due to: High humidity of atmosphere. Contaminated liquid nitrogen.	Cycle the anaerobic tent to lower humidity during grid preparation. Always use fresh liquid nitrogen and ethane/propane and use within a short amount of time.
90	CryoEM maps appear distorted or highly disordered in particular directions/particle regions, requiring revision of grid freezing conditions at step 63 (Fig. 6c).	Map distortions due to: Particles on a grid show a preferred orientation, rather than the desired random distribution of orientations. Adsorption and damage of particles at the air water interface.	Use of additives, surfactants, or sacrificial proteins may help alter preferred orientation. As above, use additives, surfactants, sacrificial proteins, or grids layered with graphene oxide, amorphous carbon, or other affinity layers to prevent protein interactions with the air water interface. Verify random distribution of particles in ice using cryoET.
90	Damaged or oxidized metallocofactor density, requiring revised anaerobic technique during protein preparation and/or freezing procedures at step 63 (Fig. 6b).	Metallocofactor density distortion due to: Exposure of particles to oxygen leading to cofactor damage, and/or oxidation. Radiation damage of cofactors or liganding groups during imaging. Partial occupancy or inhomogeneity of cofactors in protein samples	Verify anaerobicity of the solutions, buffers, and Coy tent. If necessary prepare fresh solutions, cycle the tent, and exchange catalysts in the tent. Alter dose during data collection or analyze dose fractionation of movie frames during processing. After purification, verify there is a full complement of metalloclusters using inductively coupled plasma mass-spectrometry. Damage to the metallocluster sites may occur downstream due to damage during grid preparation, see solutions above to avoid preferred orientation and interactions with the air water interface.

## Data Availability

The single particle cryo-EM maps and models have been deposited into the PDB and EMDB for release upon publication. Datasets been deposited with the following PDB and EMDB codes: 8TC3, EMD-41151 (Fe-protein, mPlum tagged); 8DBY, EMD-27317 (MoFe-protein on ultrathin carbon); 8DFC, EMD-27404 (ADP-AlF_4_^−^ stabilized MoFe-protein:Fe-protein 1:1 complex); 8DFD, EMD-27405 (ADP-AlF_4_^−^ stabilized MoFe-protein:Fe-protein 2:1 complex). All other data are available from the corresponding authors upon reasonable request.

## Key points

- Structural analysis of air-sensitive enzymes can be a challenge, because strictly anaerobic conditions are required during sample preparation and analysis. In this protocol, a customized vacuum-manifold is used in the purification of nitrogenases from *Azotobacter vinelandii* and a custom anaerobic chamber is used for protein preparation for cryoEM.
- An internal oxygen-probe made by genetically tagging the nitrogenase Fe-protein with the far-red fluorescent protein mPlum can be expressed to report on oxygen exposure.
