# Supplementary material for: Anaerobic cryoEM protocols for air-sensitive nitrogenase proteins
Source: Nat Protoc. Author manuscript; Available in PMC 2024 Nov 1. (PMC11528890; doi:10.1038/s41596-024-00973-5)
Supplement: 1 [file NIHMS2029733-supplement-1.pdf]

Supplementary information:

a

Azotobacter vinelandii strain	Name/sequence		Source
Wild-type	OP		ATCC strain 13705
Fe-protein (ΔnifH) knockout	DJ54		Gift from Dennis Dean (ref. 51)
Fe-protein-mPlum tagged	FeP-mPlum		This study
Primers			
Primer pair 1	*Forward*: 5′ -TGTTGTGTATCAGACCGCCC- 3′	*Reverse*: 5′ -CACCGGTGGTACCGATGTAG- 3′	This study
Primer pair 2	*Forward*: 5′ -ATCATCGCTCTGGCCAACAA- 3′	*Reverse*: 5′ -TGTCCAGCTTGATGTCGGTC- 3′	This study

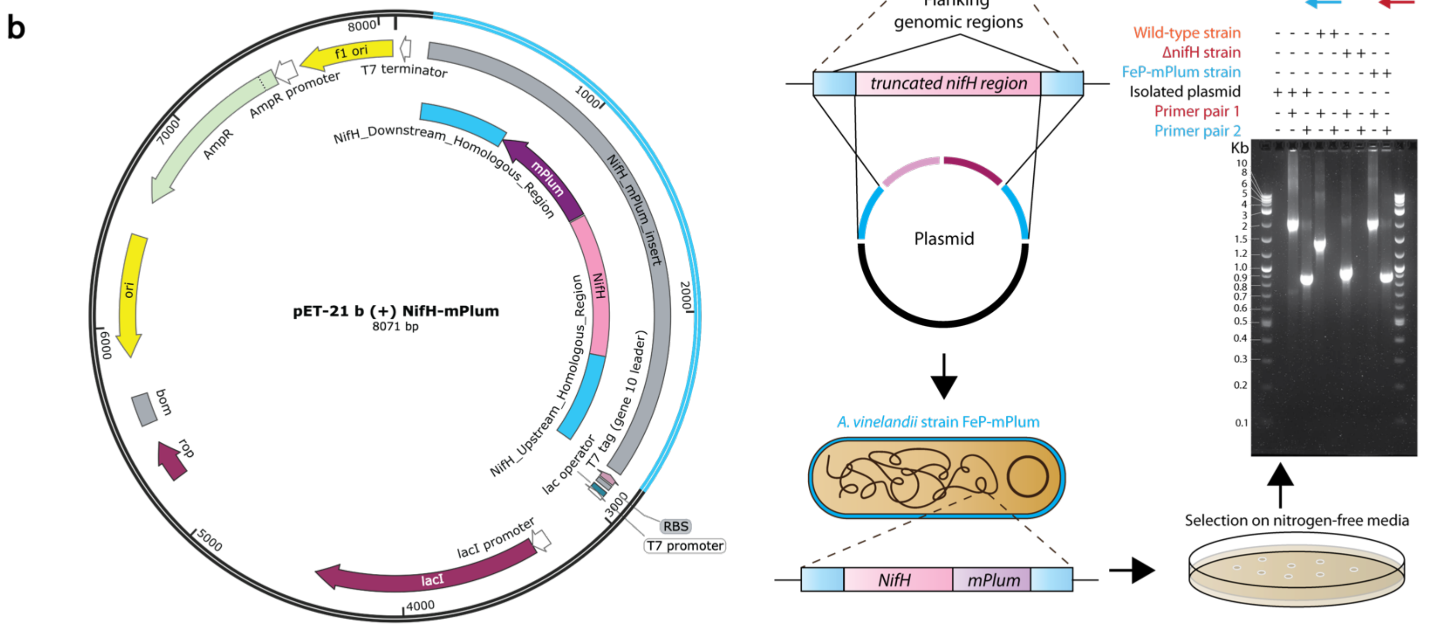

Supplementary Figure 1. Generation of mPlum-tagged Fe-protein *A. vinelandii* strain. a, Description of strains and primers utilized within this study. **b**, Plasmid map used for generation of the mPlum-tagged Fe-protein *A. vinelandii* strain. **c**, Procedure for generation of the mPlum-tagged Fe-protein *A. vinelandii* strain (FeP-mPlum) via homologous recombination.

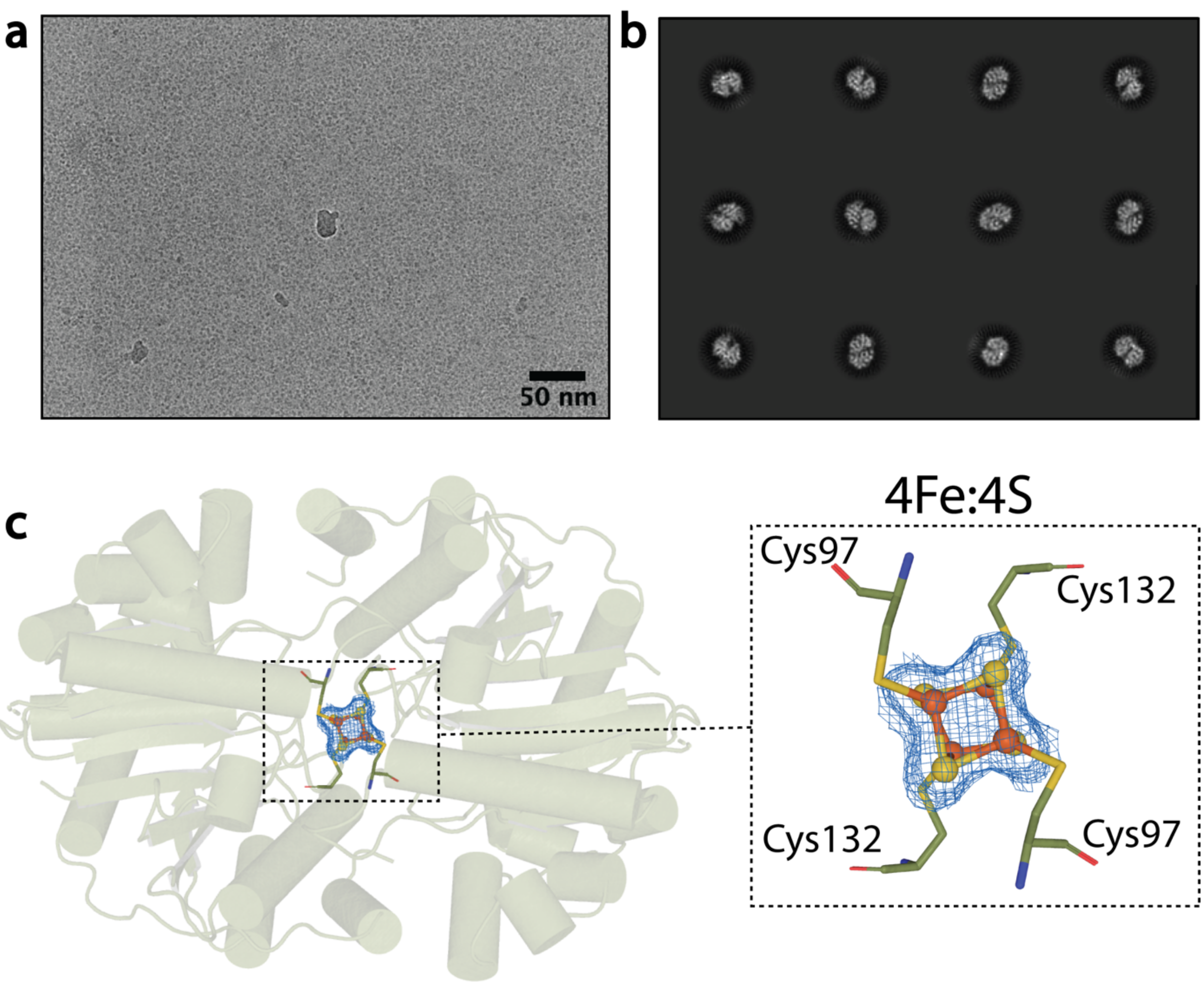

Supplementary Figure 2: Anaerobic single particle cryoEM of the nitrogenase Fe-protein. a, Representative micrograph of the mPlum-tagged Fe-protein. Scale bar represents 50 nm. **b,** 2D classes of the mPlum-tagged Fe-protein. **c,** Left panel, overview of the cryoEM structure of the Fe-protein. Right panel, zoomed view of the cryoEM density surrounding the 4Fe:4S cluster of the Fe-protein.

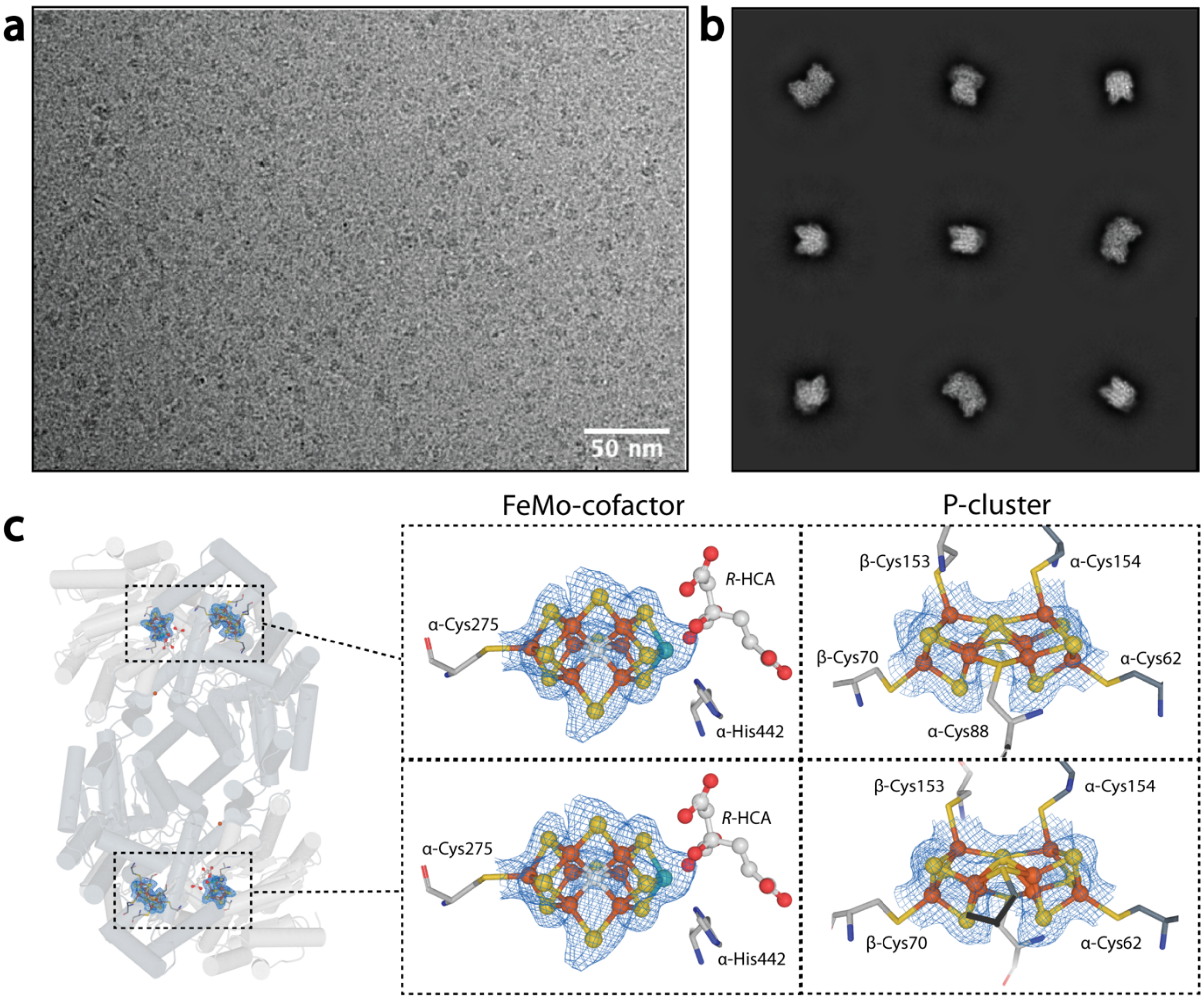

Supplementary Figure 3: Anaerobic single particle cryoEM of the nitrogenase MoFe-protein. a, Representative micrograph of the MoFe-protein on ultrathin-carbon layered grids. Scale bar represents 50 nm. **b,** 2D classes of the MoFe-protein. **c,** Left panel, overview of the cryoEM structure of the MoFe-protein. Right panel, zoomed view of the cryoEM density surrounding the FeMo-cofactors and P-clusters of the MoFe-protein.

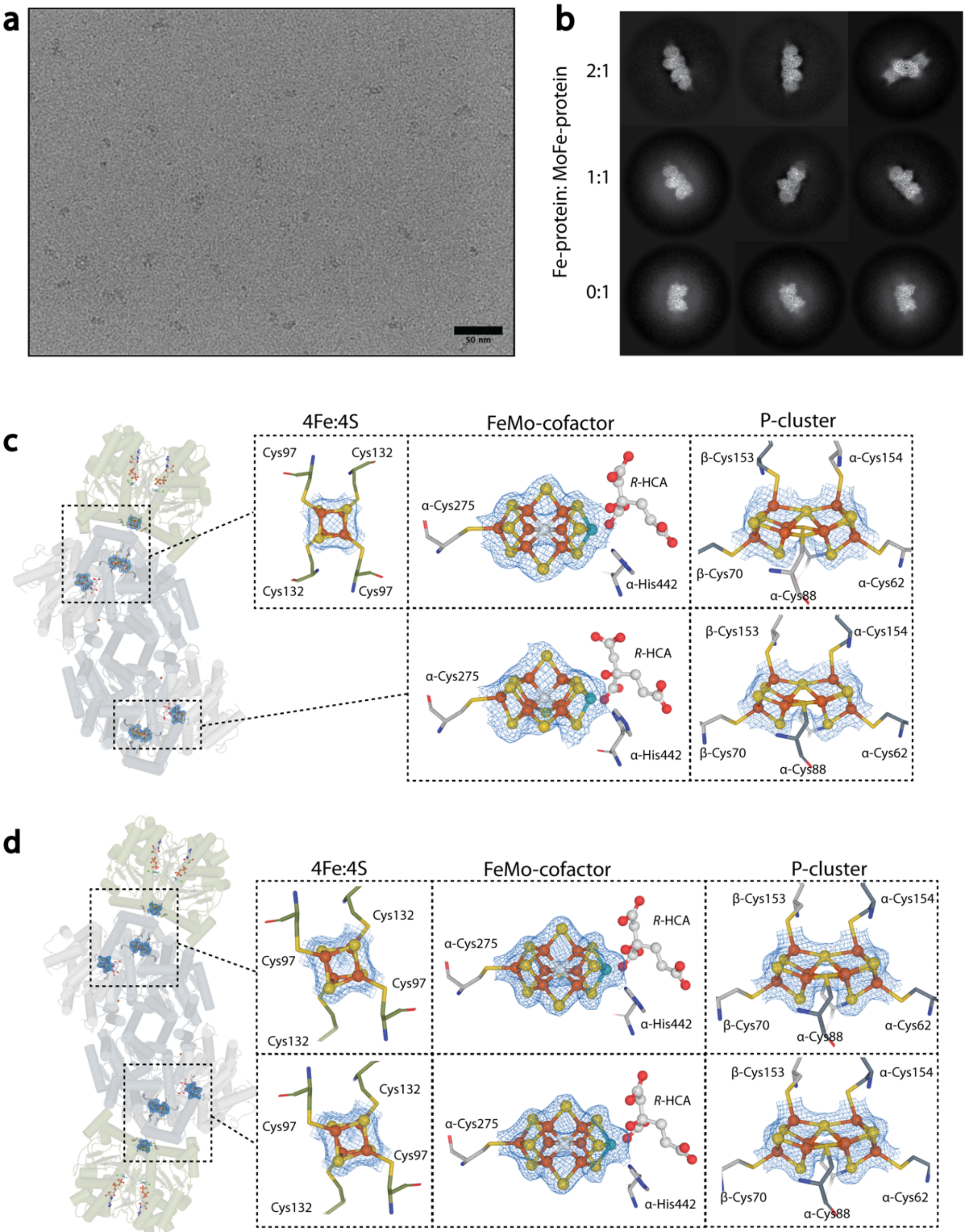

Supplementary Figure 4: Anaerobic single particle cryoEM of the ADP-AlF_4^- stabilized nitrogenase Fe-protein:MoFe-protein complex states. a, Representative micrograph of the ADP-AlF_4^- stabilized complex on ultrathin-carbon layered grids. Scale bar represents 50 nm. **b,** 2D classes of three different complex states found in the dataset. **c,** Left panel, overview of the cryoEM structure of the 1:1 ADP-AlF_4^- stabilized nitrogenase Fe-protein:MoFe-protein complex . Right panel, zoomed view of the cryoEM density surrounding the 4Fe:4S cluster, FeMo-cofactors, and P-clusters. **d,** Left panel, overview of the cryoEM structure of the 2:1 ADP-AlF_4^- stabilized nitrogenase Fe-protein:MoFe-protein complex . Right panel, zoomed view of the cryoEM density surrounding the 4Fe:4S cluster, FeMo-cofactors, and P-clusters.
